# DNA methylation subtypes for ovarian cancer prognosis

**DOI:** 10.1002/2211-5463.13056

**Published:** 2021-02-03

**Authors:** Lili Yin, Ningning Zhang, Qing Yang

**Affiliations:** ^1^ Department of Obstetrics and Gynecology Shengjing Hospital of China Medical University Shenyang China

**Keywords:** methylation, molecular subtype, ovarian cancer, prognosis, TCGA

## Abstract

Ovarian cancer is one of three major malignancies of the female reproductive system. DNA methylation (MET) is closely related to ovarian cancer occurrence and development, and as such, elucidation of effective MET subtype markers may guide individualized treatment and improve ovarian cancer prognosis. To identify potential markers, we downloaded a total of 571 ovarian cancer MET samples from The Cancer Genome Atlas (TCGA), and established a Cox proportional hazards model using the MET spectrum and clinical pathological parameters. A total of 250 prognosis‐related MET loci were obtained by Cox regression, and six molecular subtypes were screened by consensus clustering of CpG loci with a significant difference in both univariate and multivariate analyses. There was a remarkable MET difference between most subtypes. Cluster 2 had the highest MET level and demonstrated the best prognosis, while Clusters 4 and 5 had MET levels significantly lower than those of the other subtypes and demonstrated very poor prognosis. All Cluster 5 samples were at a high grade, while the percentage of stage IV samples in Cluster 4 was greater than in the other subtypes. We obtained five CpG loci using a coexpression network: cg27625732, cg00431050, cg22197830, cg03152385, and cg22809047. Our cluster analysis showed that prognosis in patients with hypomethylation was significantly worse than in patients with hypermethylation. These MET molecular subtypes can be used not only to evaluate ovarian cancer prognosis, but also to fully distinguish the tumor stage and histological grade in patients with ovarian cancer.

## Graphical abstract

Our analysis showed that ovarian cancer prognosis in patients with hypomethylation was significantly worse than in patients with hypermethylation. MET molecular subtypes can be used not only to evaluate ovarian cancer prognosis, but also to fully distinguish between the tumor stage and histological grade in patients with ovarian cancer.

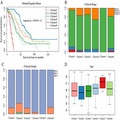

**Abbreviations**
AML: acute myeloid leukemia
CDF: cumulative distribution function
MET: DNA methylation
OS: overall survival
TCGA: The Cancer Genome Atlas

Ovarian cancer is a disease of high heterogeneity with varying molecular phenotypes, pathogeneses, and prognoses, and its morbidity is ranked number 3 among all malignant gynecological tumors. However, ovarian cancer is not easily detected at an early stage since the ovaries are located deep in the pelvis, and by the time of diagnosis, distant metastases are observed in 70% of cases. Most patients experience recurrence within 2 years, and there is a lack of effective therapies for recurrent ovarian cancer, so ovarian cancer ranks first in terms of gynecological tumor mortality. Therefore, precision therapy is urgently needed.

Epithelial ovarian cancer is the most common pathological type of ovarian cancer, and it accounts for 80–95% of ovarian malignancies. There are five histological subtypes: high‐grade serous adenocarcinoma, endometrioid adenocarcinoma, clear cell adenocarcinoma, mucous adenocarcinoma, and low‐grade serous adenocarcinoma [1, 2, 3, 4, 5]. In a large, prospective, phase III clinical study, Kommoss *et al*. [6] showed that histological grading is necessary in the prognostic evaluation of early ovarian cancer, but it has no such significance in advanced ovarian cancer.

By combining the clinicopathological and molecular biological characteristics of ovarian cancer, Shih *et al*. classified epithelial ovarian cancer into types I and II. Type I mainly includes low‐grade serous carcinoma and low‐grade endometrioid carcinoma, and most cases have an early age of onset and good prognosis. Type II mainly includes high‐grade serous carcinoma, high‐grade endometrioid carcinoma, and undifferentiated carcinoma, and most cases have rapid onset and poor prognosis. Type I epithelial ovarian cancer is significantly correlated with *BRAF*, *KRAS*, and *PTEN* mutations, while type II epithelial ovarian cancer is associated with *p53* mutations and also very frequently with *BRCA1* and *BRCA2* mutations. This dualistic theory reflects different biological behaviors and clinical prognoses of tumors, and such differences are especially remarkable between low‐ and high‐grade serous ovarian carcinomas. However, the application of this dualistic theory in nonserous ovarian carcinoma is limited. For instance, despite being type I, clear cell carcinoma has many biological behaviors similar to type II epithelial ovarian cancer. It is profoundly important to realize precision molecular typing of ovarian cancer for better clinical treatment and prognosis monitoring [7].

As a result of continuous human genome sequencing technology improvements and biomedical analysis technology advancements, new trends involving targeted molecular therapy and prognosis evaluation based on the molecular typing of malignant tumors have emerged. Targeted molecular therapy has been successfully applied in several tumors, including ER(+) and HER2(+) breast cancer and epidermal growth factor receptor (EGFR)‐mutated lung cancer [8, 9, 10, 11], thus demonstrating great progress in precision medical treatment. Using k‐means clustering, Tothill *et al*. [12] detected the gene expression spectrum of 285 cases of endometrioid and serous tumors originating from the ovary, peritoneum, and uterine tube, finally identifying six molecular subtypes, four of which (high interstitium‐reactive, high immunity, hypomethylation trix‐reactive, and interstitial low immunity) are features of high‐grade serous ovarian carcinoma and can be used to predict prognosis.

Based on gene expression, The Cancer Genome Atlas (TCGA) and Tothill *et al*. [13] divided high‐grade serous carcinoma into four subtypes: immunoreactive, differentiated, proliferative, and interstitial. Moreover, Kommoss *et al*. [14] showed that in patients with high‐grade serous ovarian carcinoma of the proliferative and interstitial molecular subtypes, bevacizumab can improve progression‐free survival to different degrees; however, this study was limited to the molecular typing of the gene expression spectrum.

In addition to gene changes, epigenetic changes, such as DNA methylation (MET), play an important role in cancer occurrence. Epigenetic inheritance refers to hereditary changes that occur under the precondition of no DNA sequence changes, including histone modification, DNA MET, RNA editing, and gene silencing. Several pathways are involved in ovarian cancer occurrence and growth, including DNA repair, cell apoptosis, cell cycle regulation, and protooncogene and tumor suppressor gene changes. Epigenetic changes in these pathways may play essential roles in ovarian cancer development, and the detection of MET signals is helpful for early diagnosis [15, 16]. DNA MET mainly occurs in CpG islands; CpG expression can be inhibited by the hypermethylation of tumor suppressor gene promoters and enhanced by the decreased demethylation probability of protooncogenes. The different regulatory effects of protooncogenes and tumor suppressor genes contribute to cancer occurrence [17, 18, 19, 20]. The tumor suppressor gene involved in ovarian cancer exists in a hypermethylated state, and an important molecular foundation for cancer occurrence is changes in this gene's MET level [21, 22].

Hu and Zhou [23] built a DNA MET interaction network for ovarian cancer, breast cancer, and glioma and confirmed that the number of DNA MET loci was associated with prognosis; however, no DNA MET molecular typing of ovarian cancer was performed.

In the present study, univariate and multivariate Cox proportional hazards models were established by analyzing Illumina Infinium^®^ HumanMethylation27 (San Diego, CA, USA) data in the TCGA database and combining the samples' MET level and clinical data. Subsequently, six molecular subtypes associated with ovarian cancer prognosis were screened by consensus clustering of MET spectra with a significant difference in both models, and patients with ovarian cancer were classified using these subtypes. Further, five CpG hypomethylation loci related to poor ovarian cancer prognosis were obtained by constructing a weighted gene coexpression network. These loci are of great significance for the clarification of ovarian cancer pathogenesis, and they can be used as effective tumor markers to provide a reference for determining clinical prognosis and individualized treatment.

## Methods

### Preprocessing of ovarian cancer expression datasets and preliminary screening of DNA MET loci

TCGA [24] GDC API was utilized to download the latest clinical follow‐up information and RNA‐Seq data. Illumina Infinium^®^ HumanMethylation27 BeadChip (Illumina 27K) microarray was acquired from UCSC Cancer Browser. Samples with complete clinical data and methylation spectrum data were selected. CpG loci with NA (Not Available) > 70% in all samples were deleted. The impute‐KNN of r package was used to fill the missing value of methylation spectrum. The unstable genomic methylation loci were further removed, involving CpGs and single nucleotide loci on sex chromosomes, as well as CpG loci that were not annotated to the gene promoter region [25]. We divided the datasets into two queues: a training set coupled with a test set. The standards for the subgroups included the following: (a) Samples were assigned to the training set and the test set randomly; and (b) the data of the two groups should be similar, including age distribution, clinical stage, follow‐up time, and mortality ratio.

### Univariate survival analysis of MET loci in the training set

The research objective was to determine the molecular subtypes of ovarian carcinoma as prognostic determinants. Therefore, CpG loci, which had an important impact on survival, were utilized as a classification feature. First and foremost, a univariate COX proportional hazards model was established based on the methylation level of each CpG loci, age, tumor grade, and stage, coupled with survival data by coxph function of the r package survival. Subsequently, we introduced the significant CpG loci obtained from the univariate model into the multivariate COX proportional hazards model, and took the significant age and clinical attributes in the univariate model as covariables. Ultimately, the CpG loci, which were still significant, were employed as classification features [25]. For each CpG island, the multivariate COX proportional hazards model formula was described below:

(1) $$h\left(t,x\right)i=h0\left(t\right)\exp\left(\beta_{\text{methy}}{\text{methy}}_{i}+\beta_{\text{age}}\text{age}+\beta_{\text{stage}}\text{stage}\right).$$

In the formula, ‘methy*_i_*’ is the carrier of the CpG locus methylation level in the sample. ‘Age’ and ‘stage’ describe the age and clinical characteristics of the patients, respectively. ‘β_methy_’, ‘β_age_’, and ‘β_stage_’ are regression coefficients. The *P*‐values of the COX regression coefficient was adjusted by Benjamini–Hochberg error detection rate. Various comparing processes were carried out.

### Screening of molecular subtypes by the consensus clustering of methylation profile with a significant difference in both univariate and multivariate analyses

Consensus ClusterPlus in the r package [26] was utilized for consensus clustering according to the method described by Zhang *et al*. [25] The subgroups of epithelial ovarian tumors were identified based on the most variable CpG loci. The algorithm is described as follows. First, double sampling of some items and features from the data matrix was conducted, in which each subsample was divided into several groups (max.) using a user‐specific clustering algorithm (k‐means, hierarchical clustering, or custom algorithms). The paired consensus value (defined as the proportion of clustering running for the combination of two items) was calculated and stored in the *k_i_* consensus matrix. Second, the final coherent sheaf clustering for each *k_i_* was completed using the distance of 1‐ consensus value and pruned into *k_i_* group through cutting, which is known as consensus clustering. The algorithm determined the ‘consensus’ clustering by measuring the stability of the clustering results applied to random data subsets from given clustering methods. In each iteration, 80% of the tumors were sampled, and a k‐means algorithm with the Euclidean squared distance measures was utilized:

(2) $$d=\sqrt{\sum_{k=1}^{N}{\left(x_{k}‐y_{k}\right)}^{2}.}$$

There were *k* = 2−10 groups, and these results were compiled for 100 times. The cluster consensus and item consensus results were obtained with Consensus ClusterPlus.r package. The graphical output results included the heat map of consensus matrix, cumulative distribution function (CDF) diagram, and Δ region diagram. The criteria of clustering number included relatively high consistency within the cluster, relatively low coefficient of variation, and insignificant increase in the area under the CDF curve (AUC). The CV (%) was calculated based on the formula below:

(3) CV(%)=(SD/MN)×100,

where SD is the standard deviation, while MN is the average value of the samples. We selected category number as the area under the CDF curve, and there was no significant change. The consensus clustering heat map was generated using the r package pheatmap.

### Clustering analysis of the methylation expression profile and analysis of the clinical characteristics of screened molecular subtypes

The stable clustering results were selected, and the methylation profile was analyzed by clustering analysis. The distance between the MET loci was calculated using the Euclidean distance. Furthermore, the distribution of various molecular subtype samples was analyzed with respect to prognosis, stage, grade, and age.

### Gene annotation of MET loci

As for the genes corresponding to the gene promoter regions annotated by the selected CpG loci, the transcription factor enrichment analysis was performed by the online tool g:profiler [27].

### WGCNA coexpression analysis of CpG loci

Based on the modification beta value of selected CpG loci, the coexpressed CpG loci were mined by WGCNA coexpression algorithm. The distance between CpG loci was calculated using the Pearson correlation coefficient. The r package *WGCNA* was used to construct weighted coexpression network and select a soft threshold of 4 to filter the CpG coexpression modules. The results showed that the coexpression network conformed to the scale‐free network. That is to say, the log(*k*) of node *k* presented in the connection is negatively correlated with the log(*P*(*k*)) of the probability of node *k*, with a correlation coefficient larger than 0.8. In order to ensure a scale‐free network, we chose β = 4. The next step was to convert the expression matrix into an adjacency matrix, and then transform the adjacency matrix into a topological matrix. Based on TOM, we utilized average‐linkage hierarchical clustering method to cluster genes. The minimum number of genes in each lncRNA network module was set at 30 according to the standard merged dynamic tree cutting. After determining the gene modules with the dynamic cutting method, we calculated eigengenes of each module in turn. The modules were clustered, and the adjacent modules were merged into new modules.

### Construction of prognosis models and data validation of independent test set

Unsupervised clustering analysis was conducted on the CpG methylation profile selected in the previous step. The similarity between samples was calculated by using the Euclidean distance. The samples were then divided into two groups according to the methylation level of CpG loci. The prognosis differences between the two groups were further analyzed. The methylation profile of 286 samples in the test set was used for validation.

## Results

### Selection of 250 characteristic MET loci

The Illumina Infinium*^®^* HumanMethylation27 BeadChip microarray contained 613 samples, with 571 samples being screened using MET detection. The missing data imputation of the MET spectrum was performed using the Impute function in the r software package, and 25 154 MET loci were selected following the exclusion of the unstable genomic MET loci. The 571 samples were assigned to either a training set (*n* = 285) or a validation set (*n* = 286). The clinicopathological information of the training and validation sets is shown in Table 1.

**Table 1:** The clinical pathological information in the training set and validation test.

	Validation	set Training set
Stage		
Stage I	5	11
Stage II	15	12
Stage III	222	215
Stage IV	41	45
Grade		
G1	3	3
G2	38	31
G3	238	243
G4	0	1
Age		
≤ 60	171	147
> 60	115	138

The MET loci and survival data were analyzed using a univariate Cox proportional hazards regression model with *P* < 0.05 as the threshold. A total of 967 loci demonstrated a significant difference in prognosis (Table S1). The 20 loci with the most significant differences are shown in Table 2.

**Table 2:** The top 20 loci with the most significant difference in prognosis.

CpGs	*P*‐value	HR	Low 95% CI	High 95% CI
cg25781123	1.42E‐05	144.7952	15.31263	1369.173
cg01278291	1.68E‐05	0.013765	0.001955	0.096903
cg21291896	2.93E‐05	1.24E+13	8 962 882	1.72E+19
cg08946332	5.46E‐05	0.21193	0.099747	0.450282
cg13804316	8.51E‐05	213 979.4	469.6279	97 496 703
cg16179125	9.40E‐05	8.255374	2.862145	23.81123
cg13060646	0.000201	4.08882	1.945923	8.591523
cg03750606	0.000282	31.81681	4.916032	205.92
cg15341340	0.000317	3 559 149	966.7441	1.31E+10
cg08013810	0.00033	0.087427	0.023115	0.330674
cg06797533	0.000383	4.351053	1.932616	9.795869
cg21022435	0.000396	0.00496	0.000263	0.093416
cg22916109	0.000475	1.07E+11	69831.7	1.65E+17
cg10415235	0.00048	6.56E+08	7370.126	5.83E+13
cg05955301	0.000514	8.98125	2.60262	30.99294
cg16016036	0.00056	0.243586	0.109202	0.543343
cg23486067	0.000569	20.19834	3.654797	111.6267
cg25634666	0.00057	0.255673	0.117688	0.555439
cg17332016	0.000602	104 162.1	141.5735	76 636 837
cg03190825	0.000654	15.75275	3.227124	76.89485

The prognostic significance of age had a log‐rank *P*‐value of 5.93e‐06, while that of stage was 0.0379. The significant MET loci were selected using a univariate Cox model followed by multivariate Cox proportional hazards regression model analysis, with stage and age as covariates. Finally, 250 significant MET loci were obtained (Table S2).

### Screening six molecular subtypes by consensus clustering of the MET loci

**Fig. 1:**
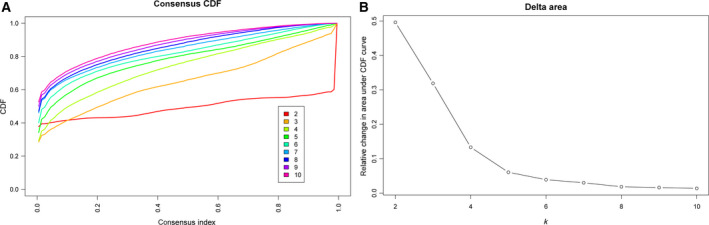
(A) Cumulative distribution function curve; different colors reflect different cluster numbers; the horizontal axis represents the consensus index; the vertical axis represents the CDF; and a larger AUC indicates better clustering. (B) CDF delta area curve of consensus clustering indicating the relative change in the area under the CDF curve for each category number *k* as compared to *k* − 1; the horizontal axis represents the category number *k*, and the vertical axis represents the relative change in the area under the CDF curve.

Consensus clustering of the MET loci with a significant difference in both the univariate and multivariate analyses was performed using the Consensus ClusterPlus function in the r software package to screen the molecular subtypes. The similarity between samples was calculated using the Euclidean distance, the clustering was performed with the k‐means function in r, and 80% sampling was conducted 100 times using a double‐sampling method. The optimal cluster number was determined by cluster dependency factors (CDFs). Different colors were used in the CDF curve to represent different cluster numbers (Fig. 1A). The area under the curve (AUC) was larger at 6 and 7 clusters, and the clustering effect was better. Further observation of the CDF delta area curve (Fig. 1B) showed that at 6 clusters, the AUC demonstrated stable clustering results. *k* = 6 was selected, and 6 molecular subtypes were obtained.

### Cluster analysis of the MET expression spectra of the 6 molecular subtypes

**Fig. 2:**
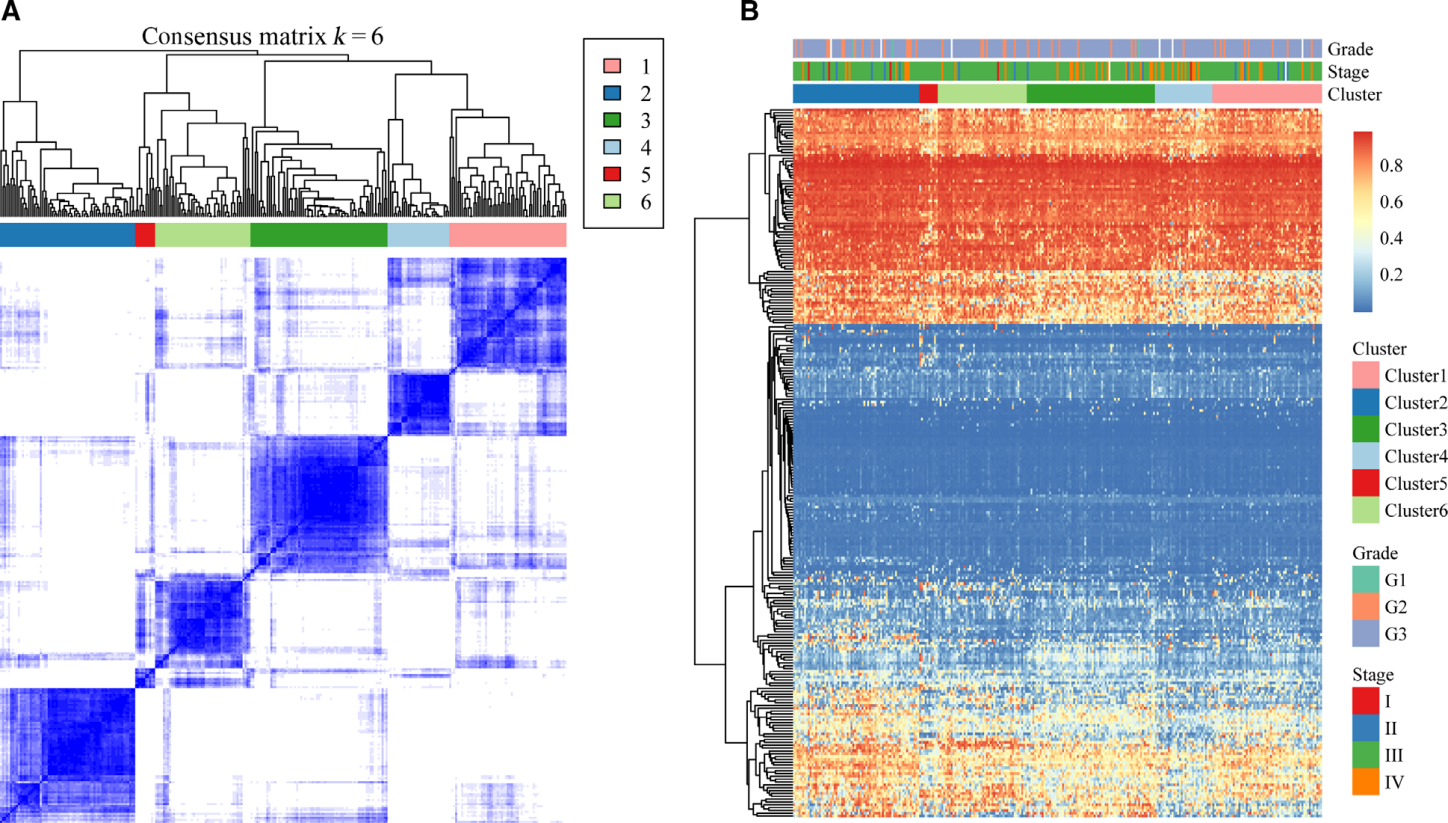
(A) Clustering heat map of samples at consensus *k* = 6. Different colors reflect different cluster numbers; the color gradient is from white to blue, indicating the consensus of progression. (B) Clustering results of 250 MET loci, clinical stage, and histological grade in six clusters of samples; red represents high expression, and blue represents low expression.

The composition and number of samples in the 6 clusters were evaluated using the consensus matrix. The color gradient was from white to blue, indicating the consensus of progression. In the matrix permutation, the same clusters were made mutually adjacent. Eventually, a color‐coded heat map was created, with dark blue blocks arranged on a diagonal white background (Fig. 2A); the heat map showed that 285 tumor samples were assigned to these 6 clusters. Furthermore, cluster analysis was performed on the 250 MET spectra; the distance between the MET loci was calculated using the Euclidean distance, and a heat map was generated by the pheatmap function in r using clinicopathological stage and histological type as notes (Fig. 2B).

The pairwise comparison of the various subtypes was performed using a *t*‐test, and the results revealed that most MET loci had a low beta value. There was a significant difference in MET level among most subtypes; the MET level in Cluster 2 was remarkably higher than in the other 5 subtypes, while the MET levels in Clusters 4 and 5 were evidently lower than in the other subtypes (Table S3).

### Analysis of the clinical characteristics of the six molecular subtypes

**Fig. 3:**
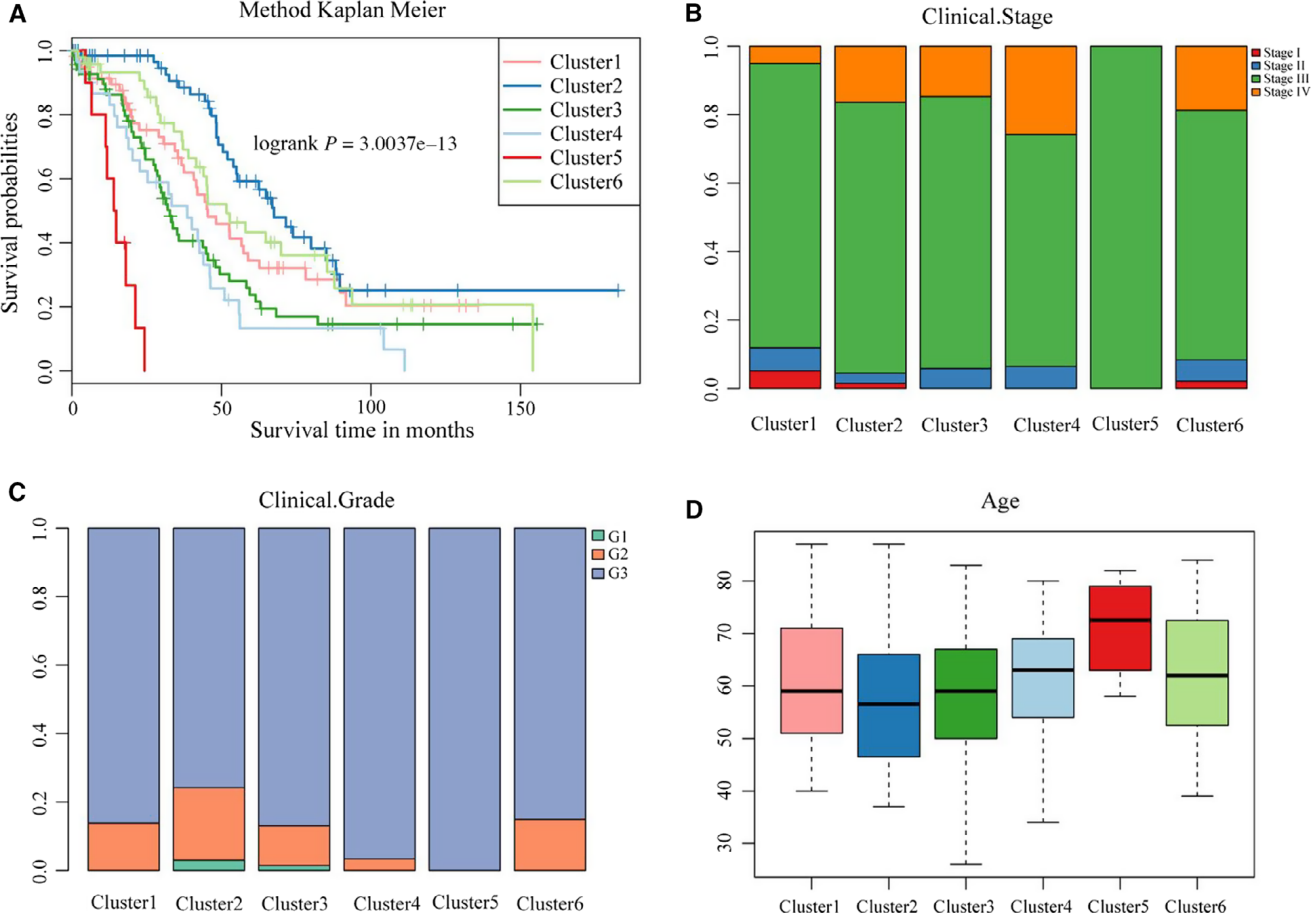
(A) Prognosis differences among the six subtypes of samples; different colors represent different molecular subtypes; the horizontal axis reflects the survival time, and the vertical axis represents the survival rate. (B) Percentages of samples of different clinical stages in the six subtypes. (C) Percentages of samples of different grades in the six subtypes; the horizontal axis represents different molecular subtypes, and the vertical axis represents the percentage. (D) Age distribution of the patients of the samples in the six subtypes; the horizontal axis represents different molecular subtypes, and the vertical axis represents age.

We further analyzed the distribution of the six molecular subtypes with respect to prognosis, stage, grade, and age (Fig. 3). There was a significant difference in prognosis among the six subtypes; the prognosis was best in Cluster 2, worst in Cluster 5, and poor in Cluster 4 (Fig. 3A), indicating that the prognosis of the hypomethylation subtypes was inferior to that of the hypermethylation subtypes. The samples in Cluster 5 were all stage III, and the percentage of stage IV samples in Cluster 4 was significantly higher than in the other subtypes (Fig. 3B). All the samples in Cluster 5 were grade 3 (Fig. 3C, Table S4), suggesting that the hypomethylation subtypes were mostly high‐grade in clinical pathology. The age of the patients of Cluster 5 was remarkably greater than the age of the patients of the other subtypes, and the age of onset was 70–80 years (Table S5), while the mean age of the patients of Cluster 2 was the lowest (Fig. 3D), indicating that the age of the patients with the hypomethylation subtypes was generally higher than the age of the patients with the hypermethylation subtypes. The above findings suggest, to a certain degree, that these DNA MET subtypes could be used to predict prognosis, tumor stage, and pathological grade in patients with ovarian cancer.

### Gene annotation and function analysis of the 250 MET loci

**Fig. 4:**
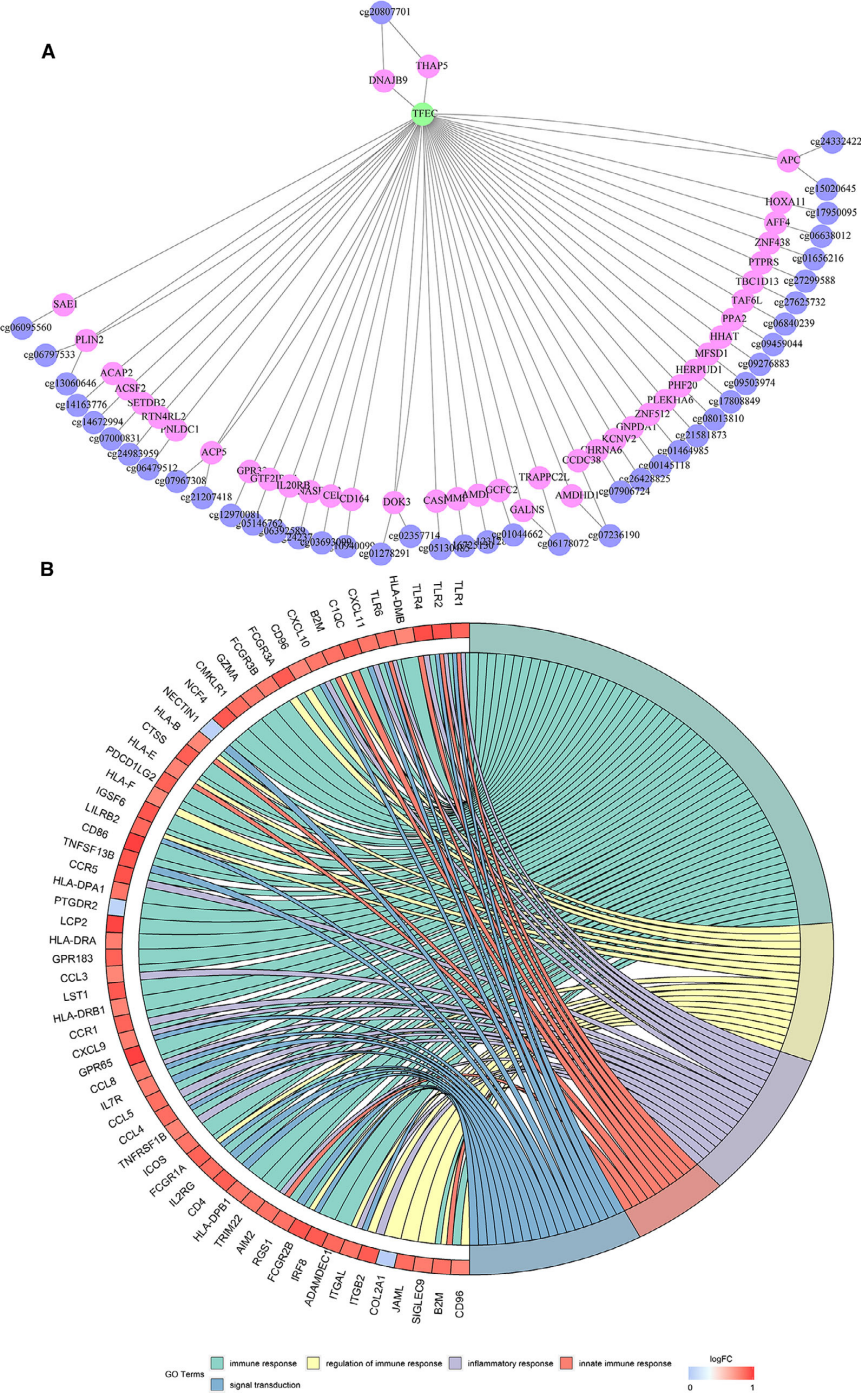
(A) Transcription factor enrichment results of genes corresponding to the gene promoter regions annotated by 250 CpG loci; green represents the transcription factors, pink represents the genes annotated by MET loci, and purple represents MET loci. (B) Chord diagram showing the top 5 enriched GO clusters for genes associated with TFEC. In each chord diagram, enriched GO clusters are shown on the right and genes contributing to enrichment are shown on the left. Positively correlated molecules are displayed in red, and negatively correlated molecules are displayed in blue. Each GO term is represented by one colored line.

A total of 285 genes corresponded to the gene promoter regions annotated by the 250 CpG loci, and these genes were subjected to transcription factor enrichment analysis using the online tool g:Profiler. It was found that 42 genes were significantly enriched to transcription factor EC (TFEC) (log‐rank *P* = 0.0107; Fig. 4A). The role of TFEC in cancer progression has been studied to a limited extent; thus, to further explore the biological functions in which TFEC may be involved, TFEC‐coexpressed molecules in the cBioPortal database were elucidated. The 300 molecules with the most positive and negative correlations according to Spearman's correlation were selected. Functional enrichment analysis was performed using the Database for Annotation, Visualization, and Integrated Discovery (DAVID) 6.7 and visualized using the GOplot function in the r software package. Finally, the five biological processes with the most significant functions were chosen: GO:0006955—immune response, GO:0050776—regulation of immune response, GO:0006954—inflammatory response, GO:0045087—innate immune response, and GO:0007165—signal transduction. TFEC may promote ovarian cancer occurrence and progression by influencing these biological functions (Fig. 4B).

### Screening five CpG loci by WGCNA

**Fig. 5:**
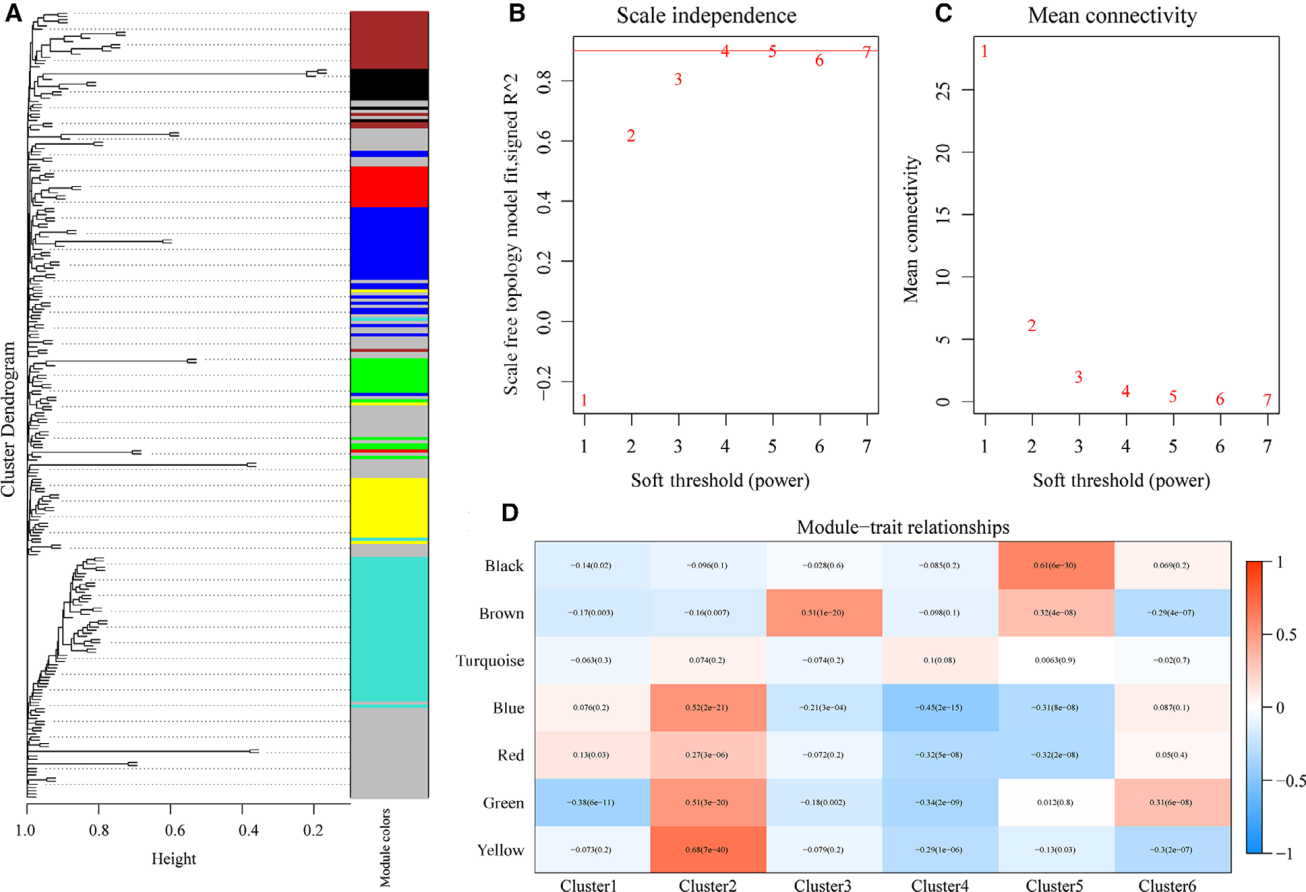
(A) Evaluation of the scale‐free model at different soft thresholds; a larger value indicates better compliance with the features of the biological network. (B) Mean connectivity at different soft thresholds; the horizontal axis represents the soft threshold, and the vertical axis represents the mean connectivity. (C) Gene dendrogram and module colors; different colors represent the genes in different modules. (D) Module–feature correlation; the row represents the eigengenes of each module, and the column represents the feature information of the samples. Red to green represents a high to low correlation coefficient. The digit in each grid indicates the correlation coefficient between gene modules and the corresponding features, and the digit in the bracket represents the *P*‐value.

Using the weighted gene coexpression network analysis (WGCNA) algorithm, the 250 significant CpG loci were mined. Evaluation of the scale‐free model was performed at different soft thresholds; a larger value and lower mean connectivity both indicated better compliance with the scale‐free distribution. Finally, β = 4 (Fig. 5A,B) was selected, and the settings of height = 0.25, deepSplit = 3, and minModuleSize = 10 were chosen. A total of seven modules were obtained (Fig. 5C); the gray module is the set of genes that could not be clustered in other modules. The statistics of the genes in the various modules are shown in Table 3. The 250 CpG loci were assigned to the seven modules. Pearson's correlation coefficient between the ME of each module and the sample characteristics was calculated; a higher correlation coefficient indicated that the module was more important. In Fig. 5D, the row represents the eigengenes of each module, and the column represents the feature information of the samples. The greatest correlations can be seen between the yellow module and Cluster 2 (*R* = 0.68, log‐rank *P* = 7e‐40), the brown module and Cluster 3 (*R* = 0.51, log‐rank *P* = 1e‐20), and the black module and Cluster 5 (*R* = 0.61, log‐rank *P* = 6e‐30).

**Table 3:** The CpG loci in different modules.

Module	Count
Black	12
Blue	34
Brown	22
Green	16
Red	14
Turquoise	49
Yellow	22

**Fig. 6:**
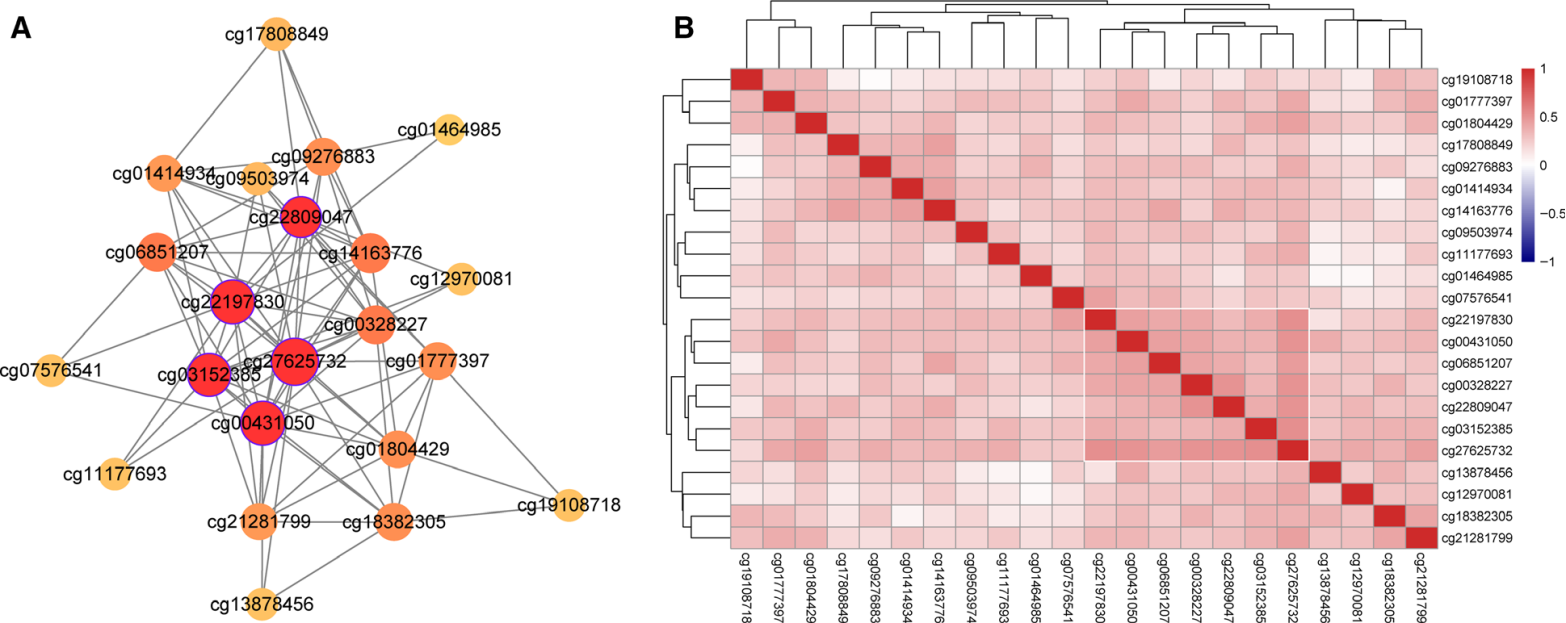
(A) Weighted interaction network of 22 CpG loci in the yellow module; the circle represents CPG loci, the connection line between two circles represents the interaction relationship, and a redder color indicates a larger node degree. (B) Correlation coefficient clustering of 22 CpG loci; a more purple color indicates a smaller correlation coefficient, and a redder color indicates a larger correlation coefficient.

Since Cluster 2 demonstrated the best prognosis of all the clusters, all the CpG loci in the yellow module, which mostly correlated with Cluster 2, were selected, and the interaction network was constructed according to their weighted relationships (Fig. 6A). In this network, the CpG loci with a network centrality > 10 were cg27625732, cg00431050, cg22197830, cg03152385, and cg22809047. Furthermore, the expression relationships among the 22 CpG loci were calculated, and a significantly higher correlation was found among 8 (cg27625732, cg00431050, cg22197830, cg03152385, cg22809047, cg00328227, cg06851207, and cg01777397) (Fig. 6B). Finally, the five CpG loci in the intersection that had a strong correlation between each other and a centrality > 10 in the weighted network were chosen as the characteristic MET loci of the Cluster 2 samples (Table 4).

**Table 4:** The annotation of five CpG loci.

CpG	Chrom	Start	End	GeneSymbol	Feature_Type
cg03152385	chr16	15 094 739	15 094 740	RP11‐72I8.1	S_Shore
cg27625732	chr9	1.29E+08	1.29E+08	TBC1D13	N_Shore
cg22197830	chr5	1.35E+08	1.35E+08	TXNDC15	N_Shore
cg22809047	chr2	1.01E+08	1.01E+08	AC016738.4	Island
cg03152385	chr16	15 094 739	15 094 740	RRN3	S_Shore
cg00431050	chr10	1.02E+08	1.02E+08	ELOVL3	N_Shore
cg22809047	chr2	1.01E+08	1.01E+08	RPL31	Island

### Cluster analysis of the five CpG loci

**Fig. 7:**
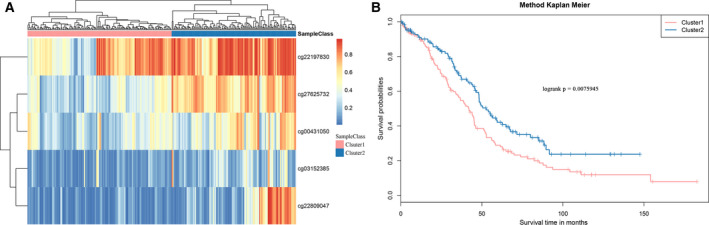
(A) MET spectrum clustering results of the five CpG loci. (B) Prognosis difference between the hypermethylation and hypomethylation groups formed by clustering. The horizontal axis represents the survival time (months), the vertical axis represents the survival rate, red indicates the hypomethylation group, and blue indicates the hypermethylation group.

Unsupervised cluster analysis was performed on the MET spectra of the five selected CpG loci, and the similarity between the samples was calculated using the Euclidean distance. Figure 7A shows that the samples were divided into two groups according to the MET level of the five CpG loci: Cluster 1 (hypomethylation group) and Cluster 2 (hypermethylation group). The prognosis difference between these two groups was further analyzed (Fig. 7B), and it was found that the prognosis in the hypomethylation group was significantly poorer than in the hypermethylation group.

### Model validation using the test dataset

**Fig. 8:**
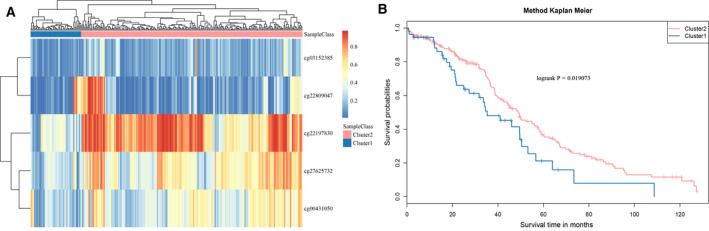
(A) Expression spectrum clustering results of the five CpG loci. (B) Prognosis difference between the hypermethylation and hypomethylation groups formed by clustering. The horizontal axis represents the survival time (months), the vertical axis represents the survival rate, red indicates the hypomethylation group, and blue indicates the hypermethylation group.

The MET spectra of the five CpG loci from the 286 samples in the test dataset were extracted and analyzed by hierarchical clustering (Fig. 8A). The results showed that the MET spectra of the five CpG loci were obviously clustered into two groups: Cluster 1 and Cluster 2. The MET level of the Cluster 1 samples was significantly higher than that of the Cluster 2 samples. The prognosis difference between Clusters 1 and 2 (Fig. 8B) was further analyzed, and it was found that the prognosis in the hypermethylation group was remarkably better than in the hypomethylation group, which was consistent with the results of the training set.

### Analysis flowchart

**Fig. 9:**
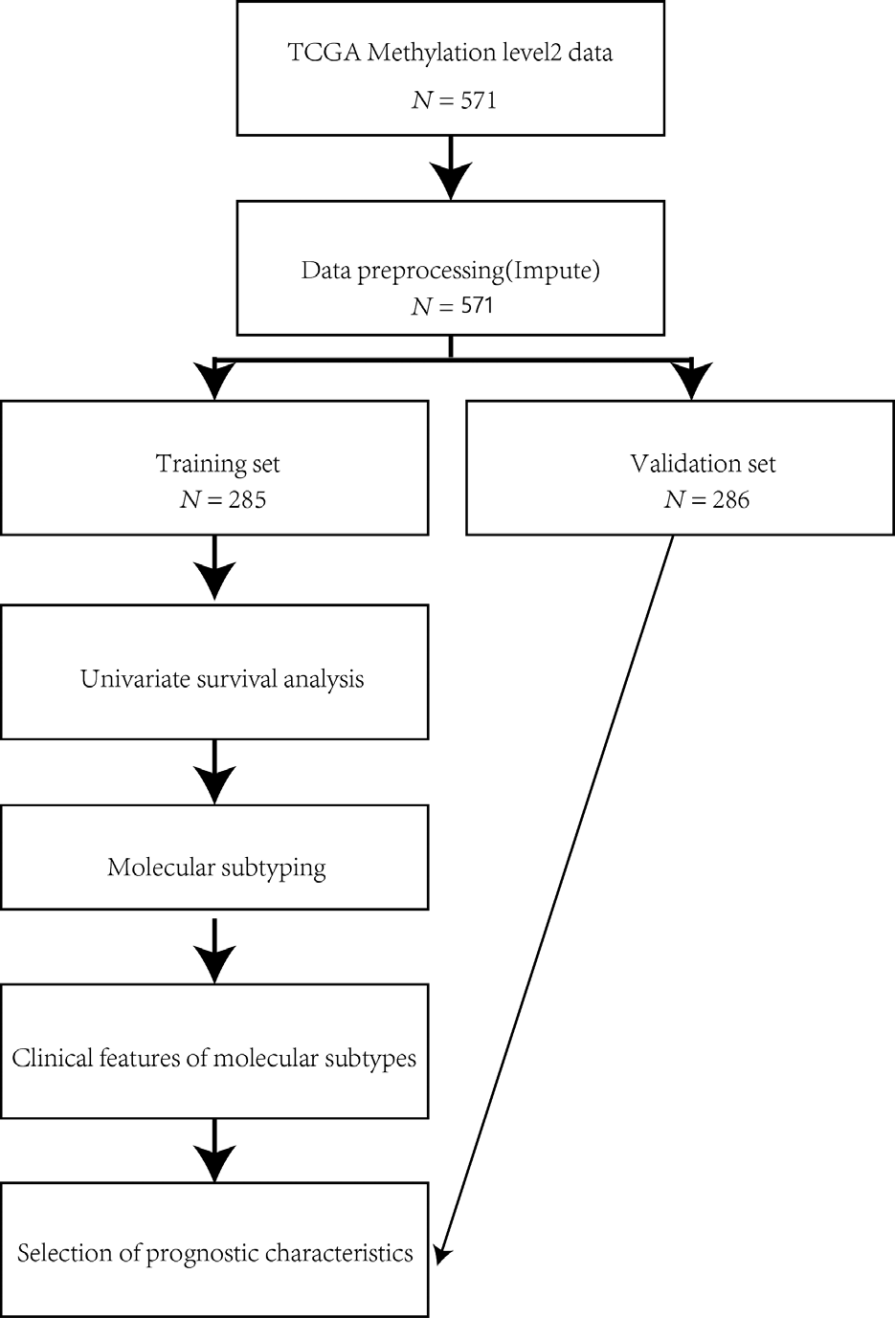
Flowchart of all the analysis.

A flowchart of the mining of subtype markers for ovarian cancer prognosis based on methylation data is shown in Fig. 9. The r packages covered in this article are listed in Table S6.

## Discussion

In recent years, an increasing number of studies have focused on exploring the molecular typing of epithelial ovarian cancer to promote the realization of personalized treatment and improve the survival rate. However, molecular typing achievements remain in the initial phase. Cancer occurrence is associated with genetic changes, and epigenetic abnormalities are also contributors. DNA MET is the major epigenetic modification mode of genomic DNA; it is an important means of regulating genomic functions [28] and is closely associated with ovarian cancer occurrence, progression, treatment, and prognosis. DNA MET‐based molecular typing and subtype markers are of great significance for guiding personalized treatment and prognostic evaluation in patients with ovarian cancer.

In the present study, 571 ovarian cancer MET samples were downloaded from the TCGA database, 250 MET loci related to ovarian cancer prognosis were screened by Cox regression analysis, and six molecular subtypes were selected by clustering with k‐means. There was a significant difference in MET loci among most subtypes. The highest MET level and best prognosis were observed in Cluster 2; Clusters 4 and 5 had remarkably lower MET levels than the other subtypes and very poor prognosis. This suggests, to a certain degree, that the prognoses of patients with a hypomethylation subtype were worse than those of patients with a hypermethylation subtype. All the samples in Cluster 5 were high‐grade, and the mean age of the patients in Cluster 5 was higher than in the other subtypes. The percentage of stage IV samples in Cluster 4 was significantly greater than in the other subtypes. The above findings suggest that these molecular subtypes can be used not only to evaluate ovarian cancer prognosis, but also to fully distinguish the tumor stage, histological grade, and age of these patients to guide subsequent treatment.

DNA MET molecular typing also plays a very important role in the diagnosis, treatment, and prognosis of other tumors. Zhang *et al*. [25] screened nine molecular subtypes by cluster analysis of DNA MET data in 669 patients with breast cancer, and the DNA MET mode was reflected in varying races, ages, tumor stages, subject states, histological types, metastatic states, and prognoses. In comparison with PAM50 subtypes using gene expression clustering, DNA MET subtypes are more precise and can be used for the precision treatment of specific histological subtypes of breast cancer.

Jurmeister *et al*. [29] constructed a DNA MET map using whole‐genome MET data from 600 cases of primary pulmonary, colorectal, and upper gastrointestinal adenocarcinoma and successfully distinguished between pulmonary enteric adenocarcinoma and metastatic colorectal cancer.

Williams *et al*. [30] measured MET levels in different histological subtypes of 154 cases of child germ cell tumors using the Illumina Infinium^®^ HumanMethylation450 BeadChip, identifying four molecular subtypes. The MET level in the germ cell tumors was low, and these molecular subtypes provided information regarding their etiology.

Also using the Illumina Infinium^®^ HumanMethylation450 BeadChip, Wu *et al*. [31] detected the DNA MET state in 482 and 421 CpG loci in 10 samples of Ewing's sarcoma, 11 samples of synovial sarcoma, and 15 samples of osteosarcoma. Moreover, they developed and validated a whole‐genome DNA MET classifier to identify osteosarcoma, Ewing's sarcoma, and synovial sarcoma. MET‐based molecular typing is of great significance for diagnosing, recognizing, and treating morphologically overlapping solid tumors.

Taskesen *et al*. [32] integrated the gene expression and DNA MET spectra of 344 samples of acute myeloid leukemia (AML) and established a regression model using Lasso. The results indicated that the subtype prediction of AML cytogenetics and molecular abnormalities could be significantly improved.

A study by Rodríguez‐Rodero *et al*. [33] demonstrated that thyroid carcinoma subtypes have promoter‐differentiated MET features, and molecular typing could be realized using abnormal DNA MET expression. Undifferentiated thyroid carcinoma was characterized by abnormal promoter hypomethylation, while differentiated papillary and follicular thyroid carcinoma was characterized by promoter hypermethylation.

To further explore the functions of the 250 screened MET loci, gene function annotation of the loci was performed, and 42 genes were found to be significantly enriched to TFEC. The *TFEC* gene is located at 7q31.2 and encodes a polypeptide with a length of 347 amino acids that is mainly localized in the nucleus and cytoplasm. According to a study by Chung *et al*. [34], TFEC is an activating transcription factor (ATF) for the nonmyosin heavy chain II‐a gene. At present, evidence for the involvement of TFEC in cancer progression is limited; however, TFEC, microphthalmia‐associated transcription factor (MITF), transcription factor EB (TFEB), and transcription factor E3 (TFE3) are important members of the family of microphthalmia transcription (MIT) factors, and recent studies have proven that changes in these transcription factors are related to melanoma, sarcoma, and renal cell carcinoma. With a similar structure to TFEB, TFEC may play an important role in regulating genes related to autophagy and lysosomes [35].

Gene regulation is complex; to investigate the effects of TFEC and its relevant factors on tumor occurrence and progression, function enrichment analysis was performed, and these genes were found to be remarkably enriched to the following biological functions: GO:0006955—immune response, GO:0050776—regulation of the immune response, GO:0006954—inflammatory response, GO:0045087—innate immune response, and GO:0007165—signal transduction. Currently, there are no reports of TFEC in ovarian cancer; thus, further investigation is needed.

Finally, five CpG loci (cg27625732, cg00431050, cg22197830, cg03152385, and cg22809047) were screened via WGCNA. The results showed that hypomethylation of these five CpG loci was associated with poor ovarian cancer prognosis. The gene annotated by the cg22809047 locus was *RPL31*, and Maruyama *et al*. [36] have shown that in comparison with benign prostate tissues, RPL31 is overexpressed in prostate cancer. In RPL31 siRNA‐treated LNCaP and BicR cells, there is an increase in the protein expression of the tumor suppressor p53 and its targets, p21 and MDM2. In addition, cell growth and cell cycle inhibition by RPL31 could be recovered by p53 siRNA treatment. RPL31 could be used as the molecular treatment target for advanced prostate cancer, and we presume that RPL31 could also be used as a target for ovarian cancer treatment. *ELOVL3* was the gene corresponding to the gene promoter region annotated by the cg00431050 locus. ELOVL3 is a member of the family of elongases of very long‐chain fatty acids (ELOVL), which includes seven members (ELOVL1–7). The proteins encoded by *ELOVL1–7* genes are involved in the elongation of fatty acid chains of different lengths, and they play an important role in regulating the biological synthesis of lipids, fatty acid metabolism, and certain metabolic diseases. There are limited studies of ELOVL3 involvement in tumors, while ELOVL2 involvement in tumors has been widely described. A study by Kang *et al*. revealed that patients with breast cancer with low ELOVL2 expression have poor prognoses. ELOVL2 expression has been correlated with the malignant phenotype of breast cancer, and its downregulation induces lipid metabolism reprogramming; thus, ELOVL2 is a novel prognostic biomarker [37]. We suggest that ELOVL3 expression may also be involved in ovarian cancer occurrence and progression by inducing lipid metabolism reprogramming.

Zhang *et al*. [38] investigated the molecular typing of serous ovarian cancer using the multi‐omics data of DNA MET and protein, miRNA, and gene expression, mainly discussing the relationship between molecular typing based on RNA sequencing data and that based on other omics data. They screened nine molecular subtypes based on RNA sequencing data; these subtypes had significant overlap with the molecular subtypes of other omics, but the functional analysis results showed that the subtypes based on an omics dataset could not be completely substituted by other omics data.

In the present study, the significance of MET in the molecular typing of ovarian cancer was analyzed using MET data, and the markers of subtypes closely related to ovarian cancer prognosis prediction were further screened. A MET data‐based ovarian cancer prognosis prediction model was subsequently developed to provide a reference for clinical trials and researchers. The study by Zhang *et al*. and our study had different focal points, despite both involving molecular typing.

Ovarian carcinoma subtyping based on MET profiles has been reported in a TCGA seminal article [39], and four subtypes were identified as significantly associated with differences in age, BRCA inactivation events, and survival based on consensus clustering of variable DNA MET data. The cluster associated with the worst prognosis was characterized by hypomethylation and associated with old age, which is in accordance with the present findings; however, our approach was different from that of the TCGA paper.

First, the samples included in the TCGA paper were 489 cases of high‐grade serous ovarian cancer, while the present paper included 571 cases of methylated ovarian cancer, including different clinical stages and grades. Our sample size was larger, and the results were more abundant. Second, a multivariate Cox proportional hazards model was used to show that 250 CpG loci were significant predictors of prognosis, and six molecular subtypes were clustered based on the methylation level at these 250 CpG loci. Clusters characterized by hypomethylation were associated with worse prognosis, stage, and grade, as well as older age. Third, WGCNA was applied to identify the five most significant CpG loci, and hypomethylation of these five loci was demonstrated to be associated with worse outcomes. Nevertheless, the present study has certain limitations. Only internal validation was performed on the MET prognostic loci, and no suitable external datasets were obtained. Thus, our study results need to be further validated with a larger sample size.

## Conclusion

We identified six molecular subtypes using ovarian cancer MET data in the TCGA database and showed that DNA MET molecular typing could accurately support the distinction of tumor stage and pathological grade in ovarian cancer. The specific CpG loci and genes can be used in clinical practice as biomarkers for individualized treatment and ovarian cancer prognosis prediction.

## Conflict of interest

The authors declare no conflict of interest.

## Author contributions

QY designed and conceptualized this study. LY and NZ were major contributors in experiment. LY analyzed the data. LY and QY were major contributors in writing the manuscript. All authors read and approved the final manuscript.

## Supporting information


**Table S1.** A total of 967 loci demonstrated a significant difference in prognosis

**Table S2.** 250 significant MET loci

**Table S3.** Distribution of methylation levels in each subtype

**Table S4.** The distribution of grade and stage in each subtype

**Table S5.** Age distribution of samples in each subtype

**Table S6.** The R package covered in this article

## Data Availability

All data during this study are included within this published article and additional files. Any material described in the article can be requested directly from corresponding author on reasonable request. All data in the current study are based on public data available in The Cancer Genome Atlas (TCGA) datasets.
