# Supplementary material for: DNA methylation subtypes for ovarian cancer prognosis
Source: FEBS Open Bio. 2021 Feb 3;11(3):851–65. doi: 10.1002/2211-5463.13056 (PMC7931230; doi:10.1002/2211-5463.13056)
Supplement: Supplementary file 1 — Table S1. A total of 967 loci demonstrated a significant difference in prognosis [file FEB4-11-851-s001.docx]

|  | **p.value** | **HR** | **Low 95%CI** | **High 95%CI** |
| --- | --- | --- | --- | --- |
| cg25781123 | 1.42E-05 | 144.7951562 | 15.31262693 | 1369.173124 |
| cg01278291 | 1.68E-05 | 0.013764938 | 0.00195529 | 0.096903013 |
| cg21291896 | 2.93E-05 | 1.24E+13 | 8962881.959 | 1.72E+19 |
| cg08946332 | 5.46E-05 | 0.21192965 | 0.099746719 | 0.450282246 |
| cg13804316 | 8.51E-05 | 213979.3831 | 469.6279451 | 97496703.26 |
| cg16179125 | 9.40E-05 | 8.255374417 | 2.862145305 | 23.81123231 |
| cg13060646 | 0.000201403 | 4.088819562 | 1.945923403 | 8.591522863 |
| cg03750606 | 0.000281975 | 31.81681312 | 4.916032377 | 205.9200427 |
| cg15341340 | 0.000317299 | 3559148.685 | 966.7441031 | 13103301403 |
| cg08013810 | 0.000330251 | 0.087426764 | 0.023114703 | 0.330674341 |
| cg06797533 | 0.000383453 | 4.351052568 | 1.932616442 | 9.795869493 |
| cg21022435 | 0.000396056 | 0.004959625 | 0.000263316 | 0.093415713 |
| cg22916109 | 0.000474596 | 1.07E+11 | 69831.69886 | 1.65E+17 |
| cg10415235 | 0.000480214 | 655605319.9 | 7370.126301 | 5.83E+13 |
| cg05955301 | 0.000513647 | 8.981249957 | 2.602620017 | 30.99294183 |
| cg16016036 | 0.000560077 | 0.243586244 | 0.10920225 | 0.543342819 |
| cg23486067 | 0.000569289 | 20.19833806 | 3.654796967 | 111.6266825 |
| cg25634666 | 0.000570325 | 0.255672953 | 0.117688191 | 0.555439406 |
| cg17332016 | 0.000602299 | 104162.1194 | 141.5735237 | 76636837.41 |
| cg03190825 | 0.000653614 | 15.75275235 | 3.22712391 | 76.89484928 |
| cg03770548 | 0.000704705 | 0.251389945 | 0.113089348 | 0.558822788 |
| cg01804429 | 0.000723359 | 0.187971669 | 0.071323146 | 0.495398062 |
| cg22227689 | 0.000728615 | 20.38850918 | 3.546430797 | 117.2139908 |
| cg02144933 | 0.000830123 | 109.7090711 | 6.980875939 | 1724.150434 |
| cg03752628 | 0.00085881 | 127.1528264 | 7.360751273 | 2196.493355 |
| cg25000382 | 0.00086465 | 289.5815017 | 10.31239604 | 8131.713114 |
| cg22197830 | 0.000917332 | 0.370511666 | 0.205986494 | 0.666446096 |
| cg17820591 | 0.000985206 | 395.9276712 | 11.28091534 | 13895.92211 |
| cg23686014 | 0.00099446 | 416.5711257 | 11.48265953 | 15112.48351 |
| cg05322019 | 0.001108745 | 0.097532205 | 0.024080383 | 0.395032382 |
| cg23146358 | 0.001141042 | 2665.955754 | 23.00829335 | 308902.5325 |
| cg27305303 | 0.001177301 | 3.208968233 | 1.586570592 | 6.490399594 |
| cg11094938 | 0.001208426 | 0.038826351 | 0.005430368 | 0.277602812 |
| cg00292662 | 0.001210467 | 83.70731188 | 5.732428267 | 1222.329131 |
| cg04491443 | 0.001255908 | 0.311019597 | 0.152973864 | 0.632351091 |
| cg22305782 | 0.001259394 | 0.021095619 | 0.002021899 | 0.220102546 |
| cg14378057 | 0.00127607 | 0.076921929 | 0.016154052 | 0.366284769 |
| cg25203561 | 0.001312483 | 0.024958838 | 0.002627762 | 0.237062361 |
| cg09783309 | 0.001350839 | 4364.366849 | 25.93921144 | 734320.6263 |
| cg22381955 | 0.00135147 | 0.032426662 | 0.003983073 | 0.263989218 |
| cg07892051 | 0.001439646 | 0.097189128 | 0.023170422 | 0.407663118 |
| cg12556325 | 0.001451762 | 0.249960296 | 0.106472841 | 0.58681772 |
| cg12966875 | 0.00146384 | 10.73696142 | 2.48804457 | 46.33451584 |
| cg02537838 | 0.001508679 | 6.104188462 | 1.99685458 | 18.65990501 |
| cg05316065 | 0.001540597 | 5.497779282 | 1.91468003 | 15.7862288 |
| cg07426848 | 0.001630013 | 0.216309781 | 0.083448141 | 0.560706576 |
| cg08942875 | 0.001751065 | 16699442.71 | 500.281598 | 5.57E+11 |
| cg09156233 | 0.001771786 | 3999.262248 | 22.06021783 | 725019.9728 |
| cg14944362 | 0.001832659 | 7.088429144 | 2.068090897 | 24.29575402 |
| cg19342782 | 0.001843006 | 0.217875934 | 0.083508958 | 0.568441082 |
| cg21023770 | 0.001904182 | 0.119700625 | 0.03134182 | 0.457160412 |
| cg23571857 | 0.002000217 | 0.267352345 | 0.115800696 | 0.61724393 |
| cg24341944 | 0.002003571 | 36.94905038 | 3.742729356 | 364.7691815 |
| cg27299588 | 0.002034489 | 0.232086308 | 0.09175998 | 0.587010309 |
| cg09034896 | 0.002049843 | 0.221506512 | 0.084960231 | 0.577507078 |
| cg22991148 | 0.002078815 | 6.388049093 | 1.961823872 | 20.80062935 |
| cg15840985 | 0.002080957 | 11.1836536 | 2.404222805 | 52.02267763 |
| cg22006386 | 0.002114128 | 336.9726801 | 8.238817886 | 13782.38829 |
| cg15811235 | 0.00211836 | 268.5926402 | 7.583554489 | 9512.95418 |
| cg16653538 | 0.002136475 | 24466.35803 | 38.66535991 | 15481626.88 |
| cg13084525 | 0.002179967 | 9.236701846 | 2.228492074 | 38.28448035 |
| cg25384595 | 0.002190743 | 0.221530103 | 0.08444933 | 0.58112464 |
| cg05656364 | 0.002198366 | 20.80464629 | 2.981436289 | 145.1761049 |
| cg01110312 | 0.002252267 | 0.300898108 | 0.139242018 | 0.650232398 |
| cg20278498 | 0.002340137 | 29.44080198 | 3.33351295 | 260.0142355 |
| cg24063470 | 0.002383083 | 16.12956634 | 2.682103513 | 96.99957859 |
| cg10549973 | 0.002420027 | 37637.36244 | 41.58686112 | 34062947.12 |
| cg20576597 | 0.002426618 | 9.546182875 | 2.220824549 | 41.03413191 |
| cg00431050 | 0.002434237 | 0.274150239 | 0.118745653 | 0.632935619 |
| cg03693099 | 0.002444296 | 4.145025486 | 1.652335434 | 10.39815277 |
| cg10289190 | 0.002473458 | 4.99E+12 | 29842.7669 | 8.33E+20 |
| cg01275830 | 0.002486738 | 0.046058084 | 0.006269288 | 0.338371316 |
| cg00209066 | 0.002514093 | 7.33E-05 | 1.52E-07 | 0.035245352 |
| cg13665593 | 0.002581325 | 178.5744101 | 6.12797656 | 5203.809058 |
| cg20098478 | 0.002585924 | 0.30525643 | 0.141074755 | 0.660511427 |
| cg05010623 | 0.002646021 | 6906.014007 | 21.68138602 | 2199722.353 |
| cg20543571 | 0.002759177 | 0.035216795 | 0.003937374 | 0.314987273 |
| cg00672638 | 0.002769739 | 78255722.75 | 528.5330525 | 1.16E+13 |
| cg12958813 | 0.002778879 | 2.413921806 | 1.355020744 | 4.300316812 |
| cg02233558 | 0.002784442 | 43.75860372 | 3.677444643 | 520.691835 |
| cg15783800 | 0.002807128 | 0.197338812 | 0.068065477 | 0.572134487 |
| cg17169998 | 0.002833038 | 5.933572331 | 1.843333111 | 19.09979287 |
| cg26177629 | 0.002911672 | 11.41305931 | 2.297317575 | 56.70000713 |
| cg27637521 | 0.00299672 | 6.9369353 | 1.930650485 | 24.92479696 |
| cg05443740 | 0.003044441 | 21.47576128 | 2.824591467 | 163.2831962 |
| cg05130485 | 0.003069184 | 8.856441387 | 2.090184384 | 37.52614107 |
| cg11876012 | 0.003095741 | 0.069360866 | 0.011837425 | 0.406416927 |
| cg15261665 | 0.003124362 | 0.342135542 | 0.16798468 | 0.696829789 |
| cg00520708 | 0.003141598 | 0.175445334 | 0.055278257 | 0.556838561 |
| cg24642820 | 0.003145796 | 0.371499875 | 0.192549954 | 0.716760272 |
| cg03712038 | 0.003203116 | 1.92E+18 | 1336296.267 | 2.76E+30 |
| cg23749163 | 0.003209232 | 17.43152098 | 2.604628458 | 116.6607555 |
| cg07260017 | 0.003277089 | 0.30009435 | 0.13453206 | 0.669406376 |
| cg17384145 | 0.003281639 | 181.8146476 | 5.665792663 | 5834.411539 |
| cg09722397 | 0.003336662 | 51599.50894 | 36.76793199 | 72413899.27 |
| cg22468803 | 0.003354147 | 292286.3423 | 65.10882972 | 1312130877 |
| cg27022827 | 0.003407683 | 0.299249098 | 0.133455952 | 0.671008083 |
| cg04368877 | 0.003439305 | 0.022290535 | 0.001743493 | 0.284984208 |
| cg12148919 | 0.003443109 | 51.20972932 | 3.664482801 | 715.6361535 |
| cg13098960 | 0.003531083 | 0.41104294 | 0.226192367 | 0.746958443 |
| cg14672994 | 0.003539386 | 0.213709953 | 0.075762713 | 0.60282878 |
| cg04315264 | 0.003583157 | 47.6836823 | 3.539900515 | 642.3156663 |
| cg06351503 | 0.00358595 | 0.154705802 | 0.044063424 | 0.54316898 |
| cg16547341 | 0.003649293 | 0.38938394 | 0.206158143 | 0.735454105 |
| cg04432009 | 0.003706186 | 0.266784744 | 0.109299596 | 0.651183558 |
| cg09685747 | 0.003712085 | 675.4071774 | 8.284934792 | 55060.76593 |
| cg18414381 | 0.003721958 | 3.320862426 | 1.475918573 | 7.472043144 |
| cg18910313 | 0.00378043 | 0.254342671 | 0.100694681 | 0.642439038 |
| cg23828212 | 0.00380689 | 104.912656 | 4.488438368 | 2452.226029 |
| cg20999934 | 0.003840937 | 74.94927123 | 4.015455678 | 1398.942912 |
| cg23751724 | 0.003993249 | 22.05488135 | 2.683967746 | 181.2308632 |
| cg26776924 | 0.004014606 | 0.001299464 | 1.40E-05 | 0.120229767 |
| cg00466249 | 0.004184189 | 8.842829938 | 1.989668543 | 39.30083811 |
| cg06850526 | 0.004223766 | 7.449749393 | 1.882171347 | 29.48656407 |
| cg10003443 | 0.004231954 | 16.81715392 | 2.43130681 | 116.322903 |
| cg25985778 | 0.004302787 | 2175933.56 | 97.03719507 | 48792495030 |
| cg21248774 | 0.004310984 | 2237161.131 | 97.67726582 | 51239046078 |
| cg06792598 | 0.004327903 | 19.53397434 | 2.5357489 | 150.4786824 |
| cg25658980 | 0.004333545 | 49.31471675 | 3.387266133 | 717.9658145 |
| cg27433088 | 0.004340657 | 34.03456976 | 3.014750524 | 384.2281241 |
| cg00269932 | 0.004425973 | 0.369820312 | 0.18641761 | 0.733659567 |
| cg25226247 | 0.00449678 | 0.364969129 | 0.182081782 | 0.73155295 |
| cg22862656 | 0.004526898 | 0.40148234 | 0.213815785 | 0.753864217 |
| cg17718515 | 0.00452771 | 1007.616894 | 8.507337267 | 119343.0769 |
| cg11456838 | 0.00456651 | 0.3696344 | 0.185811241 | 0.735313909 |
| cg24206694 | 0.004575552 | 13328.63771 | 18.77702318 | 9461168.658 |
| cg27026509 | 0.004709306 | 45.2865605 | 3.218012704 | 637.3102753 |
| cg02376703 | 0.004725176 | 128.6987777 | 4.426584145 | 3741.796123 |
| cg01414934 | 0.004739027 | 0.047096759 | 0.005650412 | 0.392556308 |
| cg25044651 | 0.004742262 | 2.19040529 | 1.271132223 | 3.774489584 |
| cg23213217 | 0.004760717 | 9.065833547 | 1.961761625 | 41.89568032 |
| cg22467071 | 0.004790631 | 0.287775285 | 0.121112233 | 0.683784063 |
| cg27488807 | 0.004850619 | 0.398364586 | 0.209966586 | 0.755807612 |
| cg14799446 | 0.00485931 | 0.363591904 | 0.179812839 | 0.735203747 |
| cg16928795 | 0.00487528 | 130.1016427 | 4.388159505 | 3857.297667 |
| cg25856383 | 0.004945806 | 3.416263542 | 1.450351312 | 8.046916972 |
| cg20880234 | 0.004946854 | 3.394418551 | 1.447508398 | 7.959938136 |
| cg15448599 | 0.005015518 | 0.002532544 | 3.89E-05 | 0.164863521 |
| cg19464252 | 0.005065848 | 0.151632684 | 0.040544744 | 0.567088809 |
| cg12228229 | 0.005094463 | 0.113633332 | 0.024808915 | 0.520479598 |
| cg20320468 | 0.005115228 | 0.325885195 | 0.148654464 | 0.714416222 |
| cg06059810 | 0.005129519 | 0.035166745 | 0.003372887 | 0.366659157 |
| cg16717225 | 0.005264787 | 0.221340411 | 0.076741347 | 0.638398714 |
| cg08174003 | 0.005390812 | 87.44187901 | 3.750527171 | 2038.66866 |
| cg10737625 | 0.005399879 | 26816708.81 | 156.7726987 | 4.59E+12 |
| cg19310430 | 0.005425579 | 75.46096491 | 3.582435642 | 1589.521151 |
| cg13273136 | 0.005472494 | 0.038548023 | 0.003875419 | 0.383429532 |
| cg18755783 | 0.005573136 | 16.98571538 | 2.292503373 | 125.8512987 |
| cg04032566 | 0.005588183 | 0.047750584 | 0.005554032 | 0.410533855 |
| cg17527798 | 0.00564981 | 0.324591328 | 0.146303543 | 0.720143397 |
| cg15652212 | 0.005777768 | 5.146517214 | 1.607939013 | 16.47241544 |
| cg00342530 | 0.005845369 | 0.342165563 | 0.159603515 | 0.733550715 |
| cg02863947 | 0.005893052 | 0.101121947 | 0.019793876 | 0.516606674 |
| cg06290096 | 0.005940634 | 0.234069365 | 0.083183556 | 0.658645415 |
| cg13033054 | 0.005997105 | 0.14692919 | 0.037415291 | 0.576988344 |
| cg10073091 | 0.006054322 | 129555.4037 | 28.96449023 | 579488970.4 |
| cg13944141 | 0.006074786 | 8.404472621 | 1.83691853 | 38.45307174 |
| cg07705908 | 0.006079757 | 2.762133098 | 1.336633467 | 5.707906795 |
| cg23797100 | 0.00608301 | 0.274232115 | 0.108811615 | 0.691132585 |
| cg04457051 | 0.006113533 | 0.204913072 | 0.06598079 | 0.636387754 |
| cg19211800 | 0.006122633 | 18.93784929 | 2.31215323 | 155.1117509 |
| cg14377791 | 0.006135108 | 0.067819812 | 0.009898102 | 0.464687789 |
| cg15822346 | 0.006141573 | 32.79462795 | 2.701316498 | 398.134622 |
| cg10196289 | 0.006193547 | 47.28167082 | 2.989521998 | 747.7972723 |
| cg02519218 | 0.006259284 | 0.095970901 | 0.017882101 | 0.5150633 |
| cg04999691 | 0.006319813 | 0.187161257 | 0.056214676 | 0.623135074 |
| cg16949889 | 0.006541724 | 4.02E-06 | 5.19E-10 | 0.031131578 |
| cg03159785 | 0.006573193 | 54.73471668 | 3.05257826 | 981.4291249 |
| cg25788012 | 0.006580641 | 112.3333773 | 3.728413338 | 3384.492682 |
| cg06589885 | 0.006609494 | 3.424820819 | 1.408673279 | 8.326556497 |
| cg21281799 | 0.006669405 | 0.285929111 | 0.115725386 | 0.706460867 |
| cg16752583 | 0.006670997 | 5.720587311 | 1.622582078 | 20.16854471 |
| cg05836145 | 0.006696579 | 0.303077889 | 0.127883209 | 0.718281995 |
| cg02533173 | 0.006773103 | 0.155625766 | 0.040484844 | 0.598233228 |
| cg20066677 | 0.006817259 | 0.000128143 | 1.94E-07 | 0.084581676 |
| cg16986846 | 0.00683635 | 9.689503271 | 1.868875301 | 50.23688503 |
| cg02992632 | 0.00696915 | 5.82173318 | 1.619363585 | 20.92956611 |
| cg24888049 | 0.006994935 | 0.251024874 | 0.091937499 | 0.685394836 |
| cg27508002 | 0.007049777 | 0.157497844 | 0.041055676 | 0.604193467 |
| cg14156381 | 0.00713549 | 8.300752575 | 1.776442722 | 38.78678016 |
| cg18382305 | 0.007189459 | 0.395903888 | 0.20144864 | 0.778063767 |
| cg11473104 | 0.007223736 | 0.103282237 | 0.019708685 | 0.541244662 |
| cg04958389 | 0.007252907 | 4.487865996 | 1.499934367 | 13.42788167 |
| cg02868338 | 0.007273018 | 1.64E+11 | 1060.345945 | 2.53E+19 |
| cg01990225 | 0.007307769 | 0.0111153 | 0.000415115 | 0.297628219 |
| cg01656216 | 0.007472484 | 0.386819278 | 0.192876294 | 0.775777837 |
| cg06840239 | 0.007498899 | 6.377369777 | 1.639935362 | 24.80027336 |
| cg10092957 | 0.007508296 | 4.021332926 | 1.449732077 | 11.1545566 |
| cg11694641 | 0.007577675 | 9147.683505 | 11.31866088 | 7393110.758 |
| cg22429822 | 0.007579073 | 81.08533834 | 3.219495002 | 2042.193602 |
| cg12845249 | 0.007609861 | 82.91972749 | 3.233357819 | 2126.483239 |
| cg22784047 | 0.007625271 | 0.018057247 | 0.000946375 | 0.344540116 |
| cg23663476 | 0.007684278 | 0.285714856 | 0.113735547 | 0.717743757 |
| cg08724563 | 0.007725309 | 10.93735399 | 1.881555477 | 63.57809475 |
| cg09234859 | 0.007733183 | 8.391639016 | 1.753982415 | 40.14841014 |
| cg15571154 | 0.007827297 | 0.073767482 | 0.010801816 | 0.503770988 |
| cg23696886 | 0.007859159 | 9.149353258 | 1.788520093 | 46.80443085 |
| cg06614002 | 0.007895761 | 0.086089576 | 0.014097962 | 0.525708273 |
| cg01606998 | 0.007991763 | 1040865.58 | 37.23330586 | 29097635326 |
| cg24727568 | 0.008107487 | 470.0777754 | 4.942966834 | 44704.5514 |
| cg10362475 | 0.00814165 | 0.53286972 | 0.334296956 | 0.849394927 |
| cg08338368 | 0.008212031 | 8.301047139 | 1.728190433 | 39.87256398 |
| cg02100848 | 0.008246786 | 0.096617018 | 0.017063133 | 0.547077038 |
| cg20325517 | 0.008290239 | 23.60092599 | 2.257655674 | 246.7177409 |
| cg10211252 | 0.008295554 | 42.69536752 | 2.6295172 | 693.2430059 |
| cg10457895 | 0.008356523 | 57.87304421 | 2.835717742 | 1181.108118 |
| cg00830029 | 0.008363136 | 0.383419805 | 0.188037269 | 0.78181707 |
| cg14047667 | 0.00841513 | 52.33481003 | 2.756090553 | 993.7744382 |
| cg24194539 | 0.00843179 | 0.48697316 | 0.285102472 | 0.831781136 |
| cg11724134 | 0.0085323 | 43.21039764 | 2.611002906 | 715.1039397 |
| cg21639968 | 0.008667129 | 98.06092271 | 3.195140451 | 3009.553011 |
| cg11177693 | 0.00871351 | 0.474545843 | 0.271890937 | 0.828250324 |
| cg06607866 | 0.008738798 | 0.001517811 | 1.19E-05 | 0.194174335 |
| cg12920798 | 0.008767839 | 6.55E-10 | 8.89E-17 | 0.004829128 |
| cg24691461 | 0.008776744 | 2.95428462 | 1.314019999 | 6.642058435 |
| cg13064571 | 0.008794153 | 0.362247769 | 0.169473548 | 0.774300461 |
| cg17808849 | 0.008799636 | 7.11E-05 | 5.61E-08 | 0.090223657 |
| cg10453365 | 0.008819748 | 92.51512297 | 3.124281701 | 2739.525049 |
| cg18390025 | 0.008860032 | 0.501665984 | 0.299281291 | 0.840910432 |
| cg25169784 | 0.0089003 | 2.61E-07 | 3.04E-12 | 0.022347455 |
| cg00554250 | 0.008901665 | 0.19341117 | 0.056475328 | 0.662375627 |
| cg03752087 | 0.008951104 | 0.462150573 | 0.259078741 | 0.824394743 |
| cg26091679 | 0.008970941 | 17.74455367 | 2.052207139 | 153.4295339 |
| cg16293656 | 0.008972843 | 11.98523783 | 1.860374183 | 77.21345907 |
| cg09989134 | 0.00898574 | 88.17850237 | 3.061652057 | 2539.625057 |
| cg18149919 | 0.008987272 | 0.397388695 | 0.198857121 | 0.79412683 |
| cg12674192 | 0.009008905 | 68.97073498 | 2.876322864 | 1653.834603 |
| cg06529761 | 0.00906869 | 0.046045217 | 0.004561427 | 0.464802339 |
| cg12729059 | 0.00909368 | 90.34384525 | 3.064011395 | 2663.831599 |
| cg03625911 | 0.009201404 | 9.064123808 | 1.725455566 | 47.61544835 |
| cg19428336 | 0.00923698 | 25.8025936 | 2.232524604 | 298.2156772 |
| cg16050957 | 0.009268473 | 38154615730 | 408.257053 | 3.57E+18 |
| cg10484958 | 0.009318711 | 0.180569262 | 0.049694349 | 0.656116012 |
| cg05959508 | 0.009342845 | 0.154580843 | 0.037821436 | 0.631790848 |
| cg12312863 | 0.009426305 | 0.415136387 | 0.213770937 | 0.806181712 |
| cg14711201 | 0.009454452 | 58181.53502 | 14.66389473 | 230845289 |
| cg20530056 | 0.00949256 | 11.51826104 | 1.817030771 | 73.0149095 |
| cg07123548 | 0.009507071 | 0.23399186 | 0.078063518 | 0.701380009 |
| cg04703844 | 0.009513576 | 733095.146 | 27.03464066 | 19879254167 |
| cg06744574 | 0.009546679 | 30.35300195 | 2.297991015 | 400.9174629 |
| cg20790056 | 0.009618557 | 3.449329519 | 1.35110702 | 8.80601903 |
| cg09503974 | 0.009673834 | 0.027386661 | 0.001794373 | 0.417989632 |
| cg06291334 | 0.009699343 | 0.020969007 | 0.001121141 | 0.392189228 |
| cg16534233 | 0.009741574 | 545.2126442 | 4.587533562 | 64796.65453 |
| cg16883145 | 0.009746359 | 0.001058446 | 5.87E-06 | 0.190917525 |
| cg09892390 | 0.009765344 | 106.0420607 | 3.084398836 | 3645.740785 |
| cg16256610 | 0.009848464 | 1.33E+11 | 475.424603 | 3.73E+19 |
| cg05046097 | 0.009903211 | 4.571021421 | 1.440322406 | 14.50663875 |
| cg26626042 | 0.009904196 | 0.000315073 | 6.88E-07 | 0.144329193 |
| cg03534410 | 0.009907464 | 0.214189899 | 0.066410124 | 0.690818055 |
| cg04985146 | 0.009973737 | 37.84048446 | 2.386182393 | 600.0808105 |
| cg19857541 | 0.010052414 | 0.243595766 | 0.08311037 | 0.713977051 |
| cg27342801 | 0.010061756 | 0.463042582 | 0.257619873 | 0.832266667 |
| cg04972979 | 0.01010831 | 19.07522704 | 2.017161698 | 180.3842929 |
| cg09459044 | 0.010139877 | 18893211.4 | 53.62527279 | 6.66E+12 |
| cg06840042 | 0.010150803 | 61.40972922 | 2.659591779 | 1417.944992 |
| cg06095560 | 0.010157391 | 0.005381175 | 0.000100152 | 0.289132358 |
| cg00769470 | 0.0102111 | 0.430696941 | 0.226478605 | 0.819061275 |
| cg06200339 | 0.010249719 | 0.426586049 | 0.222607572 | 0.817472897 |
| cg09834162 | 0.010376588 | 111539.6754 | 15.40339961 | 807685283.9 |
| cg00729875 | 0.010378024 | 637.3376189 | 4.568817063 | 88906.87346 |
| cg23910243 | 0.010399099 | 0.364525773 | 0.168453338 | 0.788818084 |
| cg15932716 | 0.010404908 | 62.85990024 | 2.646267943 | 1493.184796 |
| cg25457331 | 0.010520207 | 399.2045957 | 4.058123583 | 39270.44261 |
| cg04454951 | 0.010595205 | 21.30726876 | 2.040376729 | 222.5077829 |
| cg00237010 | 0.010717661 | 2.51236056 | 1.238189689 | 5.097729078 |
| cg06585027 | 0.010732724 | 88.90029881 | 2.82945851 | 2793.206933 |
| cg03568064 | 0.010734992 | 3.61E-05 | 1.39E-08 | 0.093405951 |
| cg25195673 | 0.0107867 | 0.303414171 | 0.12129669 | 0.758966785 |
| cg11277126 | 0.010805455 | 0.103872678 | 0.018207624 | 0.592583261 |
| cg08738269 | 0.010864435 | 37.54313517 | 2.306289432 | 611.1492246 |
| cg05859264 | 0.01098026 | 3.153941439 | 1.301433515 | 7.643376698 |
| cg09015232 | 0.010987511 | 0.412145684 | 0.208147099 | 0.816077023 |
| cg13446852 | 0.011053027 | 129.2608482 | 3.039685199 | 5496.742518 |
| cg04109382 | 0.01106947 | 0.04302523 | 0.003798892 | 0.487292211 |
| cg07940485 | 0.011155157 | 609.7265194 | 4.306778513 | 86321.2323 |
| cg14386312 | 0.01115836 | 3.35685197 | 1.317450753 | 8.553226847 |
| cg23213170 | 0.011181455 | 96.54454491 | 2.827537226 | 3296.454974 |
| cg05621401 | 0.011243187 | 42.86173794 | 2.345527303 | 783.2475779 |
| cg14226064 | 0.011384893 | 0.026563947 | 0.001599286 | 0.441223978 |
| cg11599505 | 0.011388536 | 0.371354095 | 0.172416794 | 0.799828487 |
| cg25179963 | 0.011389941 | 1148.956819 | 4.897669637 | 269536.7122 |
| cg00766889 | 0.011465004 | 6.39E+11 | 450.2461718 | 9.08E+20 |
| cg16575408 | 0.011798332 | 0.020025977 | 0.000954221 | 0.420279925 |
| cg08871016 | 0.011806955 | 15.05231904 | 1.823596621 | 124.2447513 |
| cg14869028 | 0.011809665 | 187.6837115 | 3.189188475 | 11045.18464 |
| cg24983959 | 0.011817203 | 1.96E-12 | 1.51E-21 | 0.002553158 |
| cg10294836 | 0.011975065 | 18.1707097 | 1.89279002 | 174.438098 |
| cg17543123 | 0.011990062 | 2.24E-09 | 4.01E-16 | 0.012530656 |
| cg17518962 | 0.012032401 | 271.7612752 | 3.422357655 | 21579.91599 |
| cg08365982 | 0.012087813 | 0.016856817 | 0.000694856 | 0.408937043 |
| cg27210136 | 0.01211194 | 2.212211656 | 1.189714842 | 4.113490255 |
| cg23126947 | 0.012135982 | 0.128166435 | 0.02573709 | 0.638247559 |
| cg20044189 | 0.012140186 | 119.3650973 | 2.843525083 | 5010.691321 |
| cg22981461 | 0.012341748 | 566.8326362 | 3.950939991 | 81322.22666 |
| cg24167928 | 0.012352889 | 12.2479847 | 1.720621579 | 87.18542824 |
| cg23207527 | 0.012436444 | 2.225724933 | 1.188517762 | 4.168092085 |
| cg14587868 | 0.012488079 | 0.36931111 | 0.169031881 | 0.806893322 |
| cg05610379 | 0.012573301 | 102.2673148 | 2.700065573 | 3873.462844 |
| cg24556026 | 0.012599472 | 0.362047629 | 0.162980797 | 0.804257236 |
| cg08810582 | 0.012605275 | 0.022021438 | 0.001098794 | 0.441341626 |
| cg05194726 | 0.012645352 | 0.326631539 | 0.135553117 | 0.787057977 |
| cg07883333 | 0.012691822 | 7.08E-08 | 1.69E-13 | 0.029702465 |
| cg12862537 | 0.012758986 | 0.000328671 | 5.96E-07 | 0.181162301 |
| cg01182873 | 0.012794182 | 0.105293443 | 0.017894899 | 0.619545772 |
| cg21522303 | 0.012860785 | 2184.444266 | 5.108543226 | 934081.7017 |
| cg03156547 | 0.012907401 | 0.338580856 | 0.144176597 | 0.795115147 |
| cg00187380 | 0.012930966 | 133703.0709 | 12.13789602 | 1472784999 |
| cg05922591 | 0.012933722 | 0.440732973 | 0.230991997 | 0.840918976 |
| cg23680936 | 0.012955649 | 45.89590128 | 2.244376698 | 938.5384172 |
| cg02323334 | 0.012959534 | 94427.31189 | 11.24497768 | 792933297.4 |
| cg10936763 | 0.013006671 | 38.60730342 | 2.160342757 | 689.9478669 |
| cg14218343 | 0.013021807 | 122.6878785 | 2.754950707 | 5463.733162 |
| cg04848452 | 0.013041626 | 0.02293154 | 0.001164242 | 0.451671914 |
| cg24735489 | 0.013059256 | 0.422364731 | 0.213856141 | 0.834168078 |
| cg26556134 | 0.013226942 | 0.445340094 | 0.234844894 | 0.844505476 |
| cg23191950 | 0.013280954 | 3.933510231 | 1.330420295 | 11.62978556 |
| cg07296772 | 0.013339367 | 0.071010382 | 0.008740237 | 0.576926528 |
| cg07499372 | 0.0134043 | 0.188624666 | 0.050285316 | 0.707547801 |
| cg04310489 | 0.013437403 | 137.705154 | 2.773470073 | 6837.178315 |
| cg25123470 | 0.013502763 | 3.063649218 | 1.260201411 | 7.447973355 |
| cg03565782 | 0.013503895 | 130.9485159 | 2.737149113 | 6264.73499 |
| cg12627583 | 0.013592334 | 61.70990778 | 2.335946018 | 1630.222911 |
| cg06178072 | 0.013601357 | 0.001126908 | 5.13E-06 | 0.247452179 |
| cg20807701 | 0.013643338 | 5.293083436 | 1.40808832 | 19.89699926 |
| cg22658979 | 0.01368984 | 0.180455724 | 0.046256229 | 0.703997477 |
| cg09869791 | 0.01384577 | 25.59647946 | 1.935536514 | 338.5003364 |
| cg24991452 | 0.013857706 | 85.08697619 | 2.470918729 | 2930.000664 |
| cg25903497 | 0.013886807 | 8.079395321 | 1.529298373 | 42.68403729 |
| cg20516209 | 0.013887645 | 0.205323669 | 0.058166256 | 0.724781201 |
| cg20000468 | 0.013969901 | 0.02476133 | 0.001297233 | 0.472639585 |
| cg16114640 | 0.013972253 | 0.042922784 | 0.003486645 | 0.52840641 |
| cg20908204 | 0.013974722 | 0.525173315 | 0.314245589 | 0.877679816 |
| cg13920529 | 0.014044293 | 0.001689451 | 1.04E-05 | 0.275406789 |
| cg21743649 | 0.014097021 | 0.131131111 | 0.025897518 | 0.663977451 |
| cg05960024 | 0.014118245 | 0.339812507 | 0.143511169 | 0.804624059 |
| cg19228118 | 0.014218506 | 0.0379965 | 0.002781922 | 0.51897 |
| cg19530885 | 0.014290528 | 0.406125853 | 0.197505455 | 0.835107102 |
| cg06911113 | 0.014296559 | 379.1988731 | 3.277775713 | 43868.70791 |
| cg05467106 | 0.014324689 | 3809065.48 | 20.6132369 | 7.04E+11 |
| cg17950095 | 0.014416275 | 0.426982424 | 0.215946953 | 0.844253591 |
| cg27394486 | 0.014464551 | 0.503670988 | 0.290691177 | 0.872694063 |
| cg22534509 | 0.014477294 | 33.48765149 | 2.007258494 | 558.6837996 |
| cg01892689 | 0.014519562 | 0.262580673 | 0.089860476 | 0.767285156 |
| cg26911787 | 0.014533258 | 0.071455525 | 0.008609096 | 0.593081087 |
| cg15417900 | 0.014575939 | 69.32378565 | 2.311165372 | 2079.378358 |
| cg17285325 | 0.014589139 | 0.22152683 | 0.066092474 | 0.742507176 |
| cg04809136 | 0.014594046 | 127.0736349 | 2.603362701 | 6202.635036 |
| cg26940261 | 0.014595995 | 34729.05738 | 7.882787622 | 153005191.1 |
| cg20354892 | 0.014620852 | 21.87890122 | 1.838031254 | 260.4342651 |
| cg08475088 | 0.014671187 | 0.441851335 | 0.229290205 | 0.851465079 |
| cg17720233 | 0.014766743 | 0.443845269 | 0.231014258 | 0.852755254 |
| cg24568646 | 0.015045035 | 10.03063338 | 1.563568754 | 64.34869321 |
| cg17022635 | 0.015073367 | 61.00078533 | 2.216667404 | 1678.689281 |
| cg00655307 | 0.015085131 | 1.41E-06 | 2.71E-11 | 0.073765063 |
| cg17166101 | 0.015098889 | 22.04051819 | 1.818943884 | 267.0695047 |
| cg04254119 | 0.015125264 | 0.089413809 | 0.012747314 | 0.627177556 |
| cg21380842 | 0.015185516 | 73.01495201 | 2.286417914 | 2331.674881 |
| cg08044694 | 0.015234832 | 0.351830185 | 0.15133133 | 0.817969942 |
| cg00910067 | 0.015316842 | 0.023816761 | 0.001161229 | 0.488480719 |
| cg07603484 | 0.015331113 | 0.314025947 | 0.123113697 | 0.800985572 |
| cg21584983 | 0.015527077 | 113.1265697 | 2.456245562 | 5210.236697 |
| cg13727946 | 0.015536174 | 0.402205397 | 0.19232719 | 0.841114465 |
| cg16176600 | 0.015571539 | 8.531863141 | 1.501816728 | 48.46975486 |
| cg22658985 | 0.015626832 | 1964.073276 | 4.200307 | 918405.2104 |
| cg03962522 | 0.015633898 | 2.660689174 | 1.203409024 | 5.882677243 |
| cg14204992 | 0.015651543 | 94.54668773 | 2.363417791 | 3782.266594 |
| cg24824840 | 0.015664232 | 0.409890854 | 0.198852898 | 0.844898485 |
| cg05607472 | 0.015746035 | 107133.8567 | 8.857657502 | 1295789915 |
| cg15271616 | 0.015753404 | 0.066009662 | 0.007268728 | 0.599455043 |
| cg20456055 | 0.015816137 | 21.31584749 | 1.776314487 | 255.7910536 |
| cg00841581 | 0.015958056 | 89.76875528 | 2.315459442 | 3480.272328 |
| cg00579393 | 0.016053166 | 0.305245388 | 0.116180621 | 0.801981826 |
| cg26530497 | 0.016054398 | 0.106988264 | 0.017344799 | 0.659937794 |
| cg07576541 | 0.016114848 | 0.43614197 | 0.221870924 | 0.857344504 |
| cg25375711 | 0.016114923 | 11.57480226 | 1.574955094 | 85.06658249 |
| cg10743104 | 0.01623871 | 14811.99427 | 5.883598392 | 37289284.49 |
| cg20520365 | 0.016338542 | 0.000398975 | 6.71E-07 | 0.237316377 |
| cg07611334 | 0.016428917 | 15.69386835 | 1.655494872 | 148.7757577 |
| cg22375610 | 0.016458147 | 0.392238247 | 0.182569097 | 0.842699257 |
| cg23320056 | 0.016517388 | 132.8878358 | 2.439879709 | 7237.724402 |
| cg01294695 | 0.016757364 | 0.030382455 | 0.001735018 | 0.532036876 |
| cg06980053 | 0.016925901 | 0.341130485 | 0.141127882 | 0.824571349 |
| cg26272237 | 0.016945042 | 24.29240402 | 1.771289301 | 333.1589553 |
| cg15983538 | 0.016997891 | 3.594493812 | 1.257062416 | 10.27823726 |
| cg25629694 | 0.016999524 | 5.775526105 | 1.368281321 | 24.37854063 |
| cg26219051 | 0.017031544 | 0.248003399 | 0.078894055 | 0.77959849 |
| cg13180098 | 0.017048454 | 0.300778829 | 0.112097527 | 0.807046386 |
| cg00129774 | 0.017080709 | 8.71E-08 | 1.38E-13 | 0.055198374 |
| cg08558340 | 0.017083196 | 0.521416876 | 0.305325217 | 0.890445806 |
| cg07753644 | 0.01724227 | 0.357984555 | 0.153708059 | 0.833742502 |
| cg03801144 | 0.017308116 | 70.19987011 | 2.117939206 | 2326.800387 |
| cg04663487 | 0.017379413 | 0.44635527 | 0.229623112 | 0.867652326 |
| cg16026550 | 0.017425621 | 17.73698438 | 1.657189501 | 189.8398551 |
| cg21667836 | 0.017435035 | 0.261374851 | 0.086466517 | 0.790095573 |
| cg02423618 | 0.017478558 | 2.629174995 | 1.184623997 | 5.835236473 |
| cg23950724 | 0.017496435 | 0.426962068 | 0.21159762 | 0.861524848 |
| cg01240931 | 0.017508002 | 0.33406673 | 0.135213528 | 0.825365488 |
| cg27546682 | 0.017518789 | 77.3527352 | 2.140086873 | 2795.889138 |
| cg20587336 | 0.017529888 | 56.66761753 | 2.026017284 | 1584.990859 |
| cg15942562 | 0.017531138 | 0.294496029 | 0.107400785 | 0.807516548 |
| cg01044662 | 0.017643773 | 0.003164721 | 2.73E-05 | 0.367204711 |
| cg18105675 | 0.017665243 | 0.317962582 | 0.123393041 | 0.819334729 |
| cg05103623 | 0.017723809 | 0.166693599 | 0.037915563 | 0.732858844 |
| cg13015534 | 0.017826706 | 4.877422787 | 1.314836785 | 18.09293239 |
| cg23141855 | 0.017884382 | 105.4439069 | 2.23148597 | 4982.517328 |
| cg09577651 | 0.017919209 | 18.01923619 | 1.644615671 | 197.4278117 |
| cg22199779 | 0.017919717 | 486.4194275 | 2.899495396 | 81601.73658 |
| cg19459093 | 0.017974653 | 531.9172847 | 2.937131285 | 96330.72898 |
| cg03943509 | 0.018023619 | 0.004324489 | 4.75E-05 | 0.393563019 |
| cg19182048 | 0.018128542 | 2.99E-07 | 1.16E-12 | 0.077119289 |
| cg06638012 | 0.018147551 | 0.002538104 | 1.78E-05 | 0.361143906 |
| cg08145177 | 0.018240255 | 0.302642518 | 0.112195242 | 0.816367005 |
| cg00294382 | 0.018260762 | 0.366820582 | 0.159506928 | 0.843583041 |
| cg04706338 | 0.018379227 | 0.323884331 | 0.126884311 | 0.826745714 |
| cg15492104 | 0.018397017 | 21.79325242 | 1.681472316 | 282.4583232 |
| cg21715963 | 0.018427398 | 18.54390691 | 1.635274724 | 210.2866745 |
| cg00469635 | 0.018580886 | 47.96810061 | 1.911068042 | 1204.006674 |
| cg18996334 | 0.018601112 | 1.19E-07 | 2.02E-13 | 0.069507568 |
| cg01438829 | 0.018613469 | 53460.23209 | 6.166778126 | 463450501.5 |
| cg00016968 | 0.018698908 | 5.499799589 | 1.328207671 | 22.77339319 |
| cg12421458 | 0.018720064 | 59.7043122 | 1.974403049 | 1805.40893 |
| cg00031162 | 0.018870781 | 0.188165479 | 0.046664882 | 0.758734316 |
| cg17383958 | 0.018954312 | 1797.358936 | 3.435966188 | 940201.0868 |
| cg02966851 | 0.019031227 | 5.285867714 | 1.314349142 | 21.25797218 |
| cg03259469 | 0.019113834 | 1.34E-07 | 2.39E-13 | 0.07510743 |
| cg19868691 | 0.019116314 | 15.05485904 | 1.558242265 | 145.4515679 |
| cg18828334 | 0.019284257 | 733.6615296 | 2.919918664 | 184340.4909 |
| cg26553682 | 0.019316658 | 55.97783826 | 1.920852688 | 1631.31634 |
| cg12820608 | 0.019320562 | 3.77E+11 | 75.34943112 | 1.88E+21 |
| cg09730361 | 0.019335565 | 8.08E+14 | 260.459382 | 2.51E+27 |
| cg08831701 | 0.019442192 | 18.83971368 | 1.605778093 | 221.036028 |
| cg02082571 | 0.019442201 | 0.108719446 | 0.016907141 | 0.699108029 |
| cg02141570 | 0.019614948 | 0.285900825 | 0.099885719 | 0.818328015 |
| cg02921068 | 0.01962226 | 32.46020983 | 1.745527389 | 603.6371751 |
| cg14679230 | 0.019632806 | 0.223189533 | 0.063322825 | 0.786660536 |
| cg08936952 | 0.019723025 | 0.001994524 | 1.07E-05 | 0.37122944 |
| cg02729303 | 0.019739039 | 4.34150771 | 1.263451766 | 14.9184082 |
| cg04396791 | 0.019834608 | 0.553929364 | 0.33697827 | 0.910556458 |
| cg02357714 | 0.019836337 | 0.267017834 | 0.087909463 | 0.811044917 |
| cg22016818 | 0.019865556 | 51.81942171 | 1.868916299 | 1436.796537 |
| cg01545079 | 0.019941395 | 2.044847009 | 1.119568712 | 3.734830425 |
| cg27555365 | 0.019994421 | 351939888 | 22.19904698 | 5.58E+15 |
| cg13555543 | 0.020059208 | 2952.096176 | 3.508545018 | 2483899.105 |
| cg03924115 | 0.02006716 | 0.322219744 | 0.124034571 | 0.837069558 |
| cg12230010 | 0.020141712 | 6513.558951 | 3.953533757 | 10731273.04 |
| cg24098951 | 0.02016808 | 64.75654623 | 1.91955752 | 2184.571307 |
| cg27281093 | 0.020288254 | 5.698567676 | 1.310851897 | 24.77295386 |
| cg22284975 | 0.020385574 | 1.20E-06 | 1.20E-11 | 0.121101268 |
| cg02119229 | 0.020445322 | 6619.299432 | 3.892794297 | 11255443.17 |
| cg25885771 | 0.020464941 | 83.25609698 | 1.97890206 | 3502.739132 |
| cg01367992 | 0.020474989 | 0.467242057 | 0.245509993 | 0.889231176 |
| cg27625732 | 0.020484364 | 0.441226484 | 0.220863949 | 0.881451276 |
| cg14598387 | 0.020555094 | 0.455587767 | 0.234227934 | 0.886146285 |
| cg03375833 | 0.020574951 | 0.375720274 | 0.164073099 | 0.860383121 |
| cg15170424 | 0.020594996 | 40.94759919 | 1.767868435 | 948.4336316 |
| cg22215192 | 0.020632415 | 0.328190903 | 0.127761064 | 0.843052378 |
| cg09988116 | 0.020639435 | 3.614604064 | 1.217551177 | 10.73085286 |
| cg00476577 | 0.02078307 | 60.1781648 | 1.865872575 | 1940.867543 |
| cg09831553 | 0.020851262 | 1041.651241 | 2.870890769 | 377944.4762 |
| cg06606198 | 0.020859785 | 93.51197643 | 1.990756112 | 4392.546974 |
| cg18325289 | 0.020932851 | 152605.1874 | 6.080423989 | 3830052521 |
| cg22493172 | 0.021046797 | 36.63271594 | 1.719220827 | 780.5605054 |
| cg07551659 | 0.021058706 | 17.29964483 | 1.535334265 | 194.9267453 |
| cg21835622 | 0.021148887 | 37.35457752 | 1.720076424 | 811.2223633 |
| cg22283058 | 0.021150096 | 0.181802383 | 0.042668782 | 0.774620352 |
| cg06714705 | 0.021179572 | 0.399106539 | 0.182749689 | 0.871607663 |
| cg06938878 | 0.021206477 | 0.530281762 | 0.309157417 | 0.90956494 |
| cg11513856 | 0.021219299 | 0.153412352 | 0.031139158 | 0.755812011 |
| cg10342590 | 0.021254885 | 0.002034032 | 1.04E-05 | 0.396872806 |
| cg14458615 | 0.021276826 | 0.478359653 | 0.255396221 | 0.89597237 |
| cg23898073 | 0.021325562 | 0.117247419 | 0.018905138 | 0.727154545 |
| cg25590938 | 0.021340255 | 2730861.355 | 9.038667772 | 8.25E+11 |
| cg03000846 | 0.021357611 | 0.436716288 | 0.215677966 | 0.884286513 |
| cg23696949 | 0.021360158 | 8.648457252 | 1.377396049 | 54.30232856 |
| cg26293512 | 0.021369732 | 3.190279103 | 1.187806723 | 8.568633735 |
| cg04481779 | 0.021433218 | 18.97693518 | 1.545612448 | 232.9976504 |
| cg14056306 | 0.021491555 | 6.32E-07 | 3.28E-12 | 0.121680579 |
| cg01169610 | 0.021562016 | 0.221205535 | 0.061090205 | 0.800977651 |
| cg03257423 | 0.021604344 | 0.042452066 | 0.002865849 | 0.628846064 |
| cg23911465 | 0.021657269 | 33.69859911 | 1.674024612 | 678.3625363 |
| cg11830061 | 0.02169946 | 0.350703837 | 0.143354884 | 0.857962964 |
| cg26610808 | 0.021720653 | 0.031166051 | 0.001612077 | 0.60252885 |
| cg24331162 | 0.02174219 | 4.473694691 | 1.24436949 | 16.08360247 |
| cg00483154 | 0.021760237 | 31.93042671 | 1.657016721 | 615.2938212 |
| cg27318281 | 0.021803953 | 0.455714192 | 0.232838133 | 0.891930467 |
| cg11223252 | 0.02199573 | 0.00263188 | 1.63E-05 | 0.424406146 |
| cg16725130 | 0.022048293 | 0.359594779 | 0.14981867 | 0.863099408 |
| cg04172043 | 0.022100439 | 97.79643144 | 1.931221442 | 4952.379771 |
| cg25759381 | 0.022102143 | 0.049557653 | 0.003780972 | 0.649558087 |
| cg23118151 | 0.022162093 | 19.01057592 | 1.524651603 | 237.0390691 |
| cg03786772 | 0.022185249 | 75.95474768 | 1.857988116 | 3105.037995 |
| cg12188560 | 0.02232393 | 2.277397225 | 1.124137528 | 4.613793236 |
| cg25263140 | 0.022330876 | 0.324411262 | 0.123503951 | 0.852140079 |
| cg10016608 | 0.022449533 | 3.057885833 | 1.171186159 | 7.983927834 |
| cg06580318 | 0.022511601 | 60.25530749 | 1.782134775 | 2037.27694 |
| cg23580000 | 0.022589061 | 0.000531388 | 8.14E-07 | 0.34671222 |
| cg14031452 | 0.022662355 | 0.052576376 | 0.004175374 | 0.662042545 |
| cg12598198 | 0.022703094 | 0.34375689 | 0.137188189 | 0.861362778 |
| cg24499411 | 0.022792252 | 0.337287026 | 0.132342643 | 0.859606057 |
| cg22298088 | 0.022801304 | 0.049551869 | 0.003729818 | 0.658312971 |
| cg02762689 | 0.022871553 | 0.016249777 | 0.000467563 | 0.564747653 |
| cg12073594 | 0.022902499 | 753.591458 | 2.503113511 | 226877.48 |
| cg00877964 | 0.022934369 | 3767.389039 | 3.122905844 | 4544876.113 |
| cg21742836 | 0.022942698 | 0.381119279 | 0.165973297 | 0.875152253 |
| cg05696092 | 0.023060649 | 2.245490339 | 1.117650815 | 4.51145098 |
| cg15339605 | 0.023158253 | 0.388607746 | 0.171875644 | 0.878635135 |
| cg05646865 | 0.023220141 | 0.000262059 | 2.12E-07 | 0.324420121 |
| cg17274742 | 0.023228575 | 0.509469805 | 0.284577167 | 0.912088221 |
| cg06583518 | 0.023254129 | 0.00272993 | 1.67E-05 | 0.44727842 |
| cg04731384 | 0.02332415 | 0.317471866 | 0.11778875 | 0.85567073 |
| cg20319405 | 0.02336575 | 122.3178687 | 1.918857546 | 7797.171312 |
| cg09303642 | 0.023374441 | 0.035349024 | 0.001965629 | 0.6357016 |
| cg06491116 | 0.02340563 | 1531.177024 | 2.698140218 | 868933.002 |
| cg01040850 | 0.023439018 | 0.286409604 | 0.097130113 | 0.844542016 |
| cg11649654 | 0.023441189 | 162.1748984 | 1.988891843 | 13223.79483 |
| cg13156411 | 0.023505319 | 0.00089262 | 2.05E-06 | 0.388324794 |
| cg23111544 | 0.023564458 | 0.449109328 | 0.224600747 | 0.898034364 |
| cg26900154 | 0.023628016 | 3.737968532 | 1.193186512 | 11.71016316 |
| cg15425811 | 0.023760497 | 0.538490622 | 0.314880623 | 0.920895504 |
| cg14947494 | 0.02379104 | 2.23E-09 | 7.04E-17 | 0.070770592 |
| cg23337289 | 0.023896133 | 0.307641946 | 0.110616772 | 0.855598708 |
| cg15013019 | 0.024112183 | 0.454395719 | 0.228945977 | 0.901852359 |
| cg16178491 | 0.024173221 | 3.45144604 | 1.175596747 | 10.13313434 |
| cg26428825 | 0.024197081 | 0.049052068 | 0.003565431 | 0.674842746 |
| cg01316819 | 0.024296978 | 20166.08093 | 3.621453918 | 112294904 |
| cg24611092 | 0.024337312 | 0.431205003 | 0.207351724 | 0.896726352 |
| cg06392589 | 0.024380236 | 0.226651276 | 0.062242089 | 0.825338633 |
| cg07906495 | 0.024417608 | 869632.4311 | 5.844467793 | 1.29E+11 |
| cg05788638 | 0.024448638 | 0.410572716 | 0.189067467 | 0.891586255 |
| cg16617137 | 0.02453602 | 0.459986612 | 0.233768099 | 0.905117869 |
| cg07559730 | 0.024562792 | 7.67E-07 | 3.57E-12 | 0.164413033 |
| cg07295034 | 0.024666479 | 1623492.495 | 6.199220427 | 4.25E+11 |
| cg07519011 | 0.024693168 | 0.000430354 | 4.97E-07 | 0.372464215 |
| cg17838765 | 0.024724586 | 1884.336996 | 2.610323049 | 1360263.02 |
| cg18042806 | 0.024771214 | 0.304541181 | 0.107855229 | 0.859905747 |
| cg21207418 | 0.024786514 | 0.166611229 | 0.034844807 | 0.796655332 |
| cg05023116 | 0.024827836 | 0.529414881 | 0.303781017 | 0.922638681 |
| cg22766145 | 0.024830806 | 0.329587521 | 0.125014213 | 0.868924669 |
| cg16377880 | 0.024831469 | 5.599224854 | 1.243652601 | 25.20906476 |
| cg26674929 | 0.02496469 | 3.67E+14 | 67.90447735 | 1.98E+27 |
| cg06489804 | 0.025093519 | 3.07E+11 | 27.27822954 | 3.44E+21 |
| cg03029616 | 0.025116845 | 0.518425838 | 0.291741053 | 0.921246243 |
| cg13925920 | 0.025201572 | 0.003791163 | 2.87E-05 | 0.49993984 |
| cg19789466 | 0.025215558 | 8.079737243 | 1.296461591 | 50.35409792 |
| cg24332422 | 0.025376226 | 0.328698476 | 0.123929985 | 0.871804251 |
| cg17534899 | 0.025396568 | 33.43681495 | 1.540842892 | 725.5902596 |
| cg10940099 | 0.025414586 | 1.85E-10 | 5.39E-19 | 0.063398779 |
| cg10648908 | 0.025421297 | 26.64974645 | 1.497649487 | 474.2157573 |
| cg06385087 | 0.025439825 | 6.655897823 | 1.262385842 | 35.09305504 |
| cg20057066 | 0.025504679 | 49.99913727 | 1.615050195 | 1547.886087 |
| cg12836863 | 0.025586715 | 0.528418485 | 0.301832514 | 0.925102772 |
| cg07748017 | 0.025652413 | 19.92745643 | 1.439088611 | 275.9409787 |
| cg12385425 | 0.025665315 | 27.39583949 | 1.495526857 | 501.851249 |
| cg01091565 | 0.025678106 | 0.39895831 | 0.177968876 | 0.89435713 |
| cg18986165 | 0.025763252 | 0.54683596 | 0.321686654 | 0.929567834 |
| cg12878228 | 0.02587974 | 0.238897802 | 0.067800105 | 0.84177097 |
| cg24237576 | 0.025942664 | 0.383873429 | 0.165290079 | 0.891516361 |
| cg27550918 | 0.025986099 | 2.183905803 | 1.097991922 | 4.34378838 |
| cg21443584 | 0.026010635 | 18.53617857 | 1.417658089 | 242.364445 |
| cg03152385 | 0.026014619 | 0.263430261 | 0.081389398 | 0.852635659 |
| cg00420715 | 0.026033441 | 47.49761021 | 1.585599389 | 1422.820285 |
| cg07952391 | 0.026167963 | 3.227455463 | 1.149089962 | 9.06497238 |
| cg07026910 | 0.02630964 | 0.002782908 | 1.55E-05 | 0.50006916 |
| cg20910746 | 0.026309818 | 3.44169209 | 1.15668972 | 10.24064124 |
| cg15643724 | 0.026360309 | 6.18E-05 | 1.19E-08 | 0.320289509 |
| cg15020645 | 0.02646519 | 0.362402052 | 0.147875061 | 0.888150081 |
| cg15046693 | 0.026512637 | 0.515685888 | 0.287277588 | 0.925696772 |
| cg25620220 | 0.026554302 | 0.447425354 | 0.21982469 | 0.910677721 |
| cg18433380 | 0.026611268 | 0.013161813 | 0.000286289 | 0.605098677 |
| cg13271963 | 0.026634038 | 0.108811284 | 0.015309926 | 0.773347691 |
| cg25829729 | 0.026644982 | 0.227019642 | 0.061193004 | 0.842219121 |
| cg27662379 | 0.026728563 | 118.3007287 | 1.734062765 | 8070.678112 |
| cg11393848 | 0.026757402 | 5.991337123 | 1.228956339 | 29.20862148 |
| cg04171565 | 0.026767243 | 0.000957709 | 2.04E-06 | 0.44930797 |
| cg10409799 | 0.026772115 | 10.77287915 | 1.314591174 | 88.28214241 |
| cg20684973 | 0.026943126 | 59.61788335 | 1.59415052 | 2229.5837 |
| cg03621001 | 0.026950825 | 0.29760045 | 0.101692723 | 0.870918049 |
| cg26825412 | 0.027049391 | 2.694589024 | 1.119036276 | 6.488449179 |
| cg20641580 | 0.027146259 | 868236851.9 | 10.21364023 | 7.38E+16 |
| cg12411068 | 0.027284393 | 74.22750034 | 1.620680997 | 3399.633744 |
| cg13278334 | 0.02745389 | 24.07163139 | 1.424048576 | 406.8986462 |
| cg24670151 | 0.027586591 | 0.241331453 | 0.068134495 | 0.854792716 |
| cg16192029 | 0.027755115 | 0.23468004 | 0.064539274 | 0.853352034 |
| cg14163776 | 0.027892222 | 0.259828454 | 0.078153876 | 0.863819285 |
| cg17407908 | 0.028011692 | 0.163983019 | 0.032685324 | 0.822706566 |
| cg17969298 | 0.028188665 | 1.50E-13 | 5.31E-25 | 0.042537406 |
| cg02539714 | 0.028268135 | 8.67E-14 | 1.85E-25 | 0.040652795 |
| cg06307169 | 0.02828881 | 0.006731347 | 7.71E-05 | 0.587459851 |
| cg10106388 | 0.02844519 | 0.484848182 | 0.253732103 | 0.92648016 |
| cg23841186 | 0.028506225 | 0.516207331 | 0.285655262 | 0.932837739 |
| cg08258650 | 0.028528172 | 41.63452116 | 1.479347918 | 1171.755021 |
| cg24264506 | 0.028599601 | 23120.62536 | 2.861141699 | 186835666.8 |
| cg12087643 | 0.028754883 | 0.134942337 | 0.022414902 | 0.812380741 |
| cg00344358 | 0.028818238 | 0.010253141 | 0.000168797 | 0.622800105 |
| cg00987015 | 0.028852697 | 4.519064541 | 1.168414488 | 17.47833884 |
| cg22243733 | 0.028908852 | 0.163486796 | 0.032201917 | 0.830010586 |
| cg19298821 | 0.028926532 | 0.311874778 | 0.109640096 | 0.887137829 |
| cg05869585 | 0.029069988 | 0.19845398 | 0.046445468 | 0.847961779 |
| cg00540544 | 0.029087935 | 55.17123784 | 1.504676409 | 2022.936935 |
| cg07841014 | 0.029123068 | 0.450493802 | 0.220085506 | 0.922117362 |
| cg15298323 | 0.02924518 | 0.406527391 | 0.18099328 | 0.913097547 |
| cg19731268 | 0.02931776 | 56.10299738 | 1.499497058 | 2099.068017 |
| cg04511195 | 0.0294083 | 0.000282466 | 1.81E-07 | 0.441323092 |
| cg19713196 | 0.029445883 | 1864.247408 | 2.121689232 | 1638043.097 |
| cg20542800 | 0.029520121 | 0.1789182 | 0.037988475 | 0.842669318 |
| cg11245384 | 0.029579223 | 2.023169968 | 1.072365148 | 3.816999022 |
| cg17351116 | 0.029580546 | 85407.99831 | 3.082586205 | 2366365671 |
| cg13219107 | 0.029674924 | 8.06E-12 | 8.07E-22 | 0.080531179 |
| cg08203715 | 0.029693281 | 0.387094167 | 0.164528362 | 0.910735951 |
| cg26608667 | 0.0296987 | 0.185389644 | 0.040574751 | 0.847061771 |
| cg05659526 | 0.029733593 | 0.000850023 | 1.45E-06 | 0.499087583 |
| cg12100791 | 0.029770085 | 0.515174034 | 0.283245859 | 0.937010293 |
| cg01639034 | 0.029845878 | 10.61710233 | 1.259553293 | 89.49431716 |
| cg16129988 | 0.030095194 | 8114021.527 | 4.628060677 | 1.42E+13 |
| cg21435394 | 0.030217394 | 191.4968392 | 1.652938701 | 22185.35957 |
| cg17917518 | 0.030312261 | 0.225283687 | 0.058482491 | 0.867827947 |
| cg01351032 | 0.030482562 | 4.885103823 | 1.161136025 | 20.55249243 |
| cg18641050 | 0.030522381 | 0.201562977 | 0.047226545 | 0.86027113 |
| cg24205633 | 0.030584257 | 1794.39779 | 2.016856246 | 1596476.415 |
| cg06346081 | 0.030590933 | 0.353709266 | 0.137891714 | 0.90730793 |
| cg22585988 | 0.030647268 | 2.861844056 | 1.103063983 | 7.424910547 |
| cg06391660 | 0.030667919 | 0.000328772 | 2.28E-07 | 0.473642391 |
| cg12346515 | 0.030857725 | 1.90E-14 | 6.61E-27 | 0.054391506 |
| cg11590700 | 0.031126533 | 352.9397882 | 1.702402147 | 73171.01566 |
| cg25884854 | 0.031145022 | 95.08044327 | 1.510791222 | 5983.812033 |
| cg12970081 | 0.031149027 | 0.572211075 | 0.344406535 | 0.950694836 |
| cg05252264 | 0.031204426 | 0.481740009 | 0.247890167 | 0.936194604 |
| cg05522288 | 0.031227327 | 157.793843 | 1.57819196 | 15776.84941 |
| cg04595372 | 0.031328817 | 0.420532359 | 0.191120873 | 0.925317376 |
| cg16545079 | 0.031341128 | 2.32877427 | 1.078628705 | 5.027855809 |
| cg17976829 | 0.031393142 | 0.000526795 | 5.44E-07 | 0.50977438 |
| cg16022344 | 0.031445223 | 2.21E-19 | 2.23E-36 | 0.02188017 |
| cg16723180 | 0.031512064 | 0.087653482 | 0.009533017 | 0.805949749 |
| cg13334054 | 0.031631409 | 0.208415863 | 0.049863465 | 0.871122203 |
| cg16509658 | 0.031674656 | 0.000282556 | 1.64E-07 | 0.488188205 |
| cg17055959 | 0.031697317 | 0.026299121 | 0.000951349 | 0.727013846 |
| cg21581873 | 0.031752337 | 0.350836102 | 0.134876512 | 0.912582693 |
| cg07906724 | 0.031905385 | 0.470695845 | 0.236479881 | 0.936885528 |
| cg24247865 | 0.031962843 | 3661.772468 | 2.02874773 | 6609287.792 |
| cg26322315 | 0.031996821 | 0.451753084 | 0.2185199 | 0.933923402 |
| cg11979312 | 0.032007227 | 0.353239308 | 0.136455952 | 0.914419681 |
| cg17469356 | 0.032146801 | 60.81898607 | 1.419244691 | 2606.280009 |
| cg22325715 | 0.032234194 | 0.532504269 | 0.299119863 | 0.947983842 |
| cg11304234 | 0.032240693 | 3.464788091 | 1.111037582 | 10.8049959 |
| cg01100796 | 0.032241348 | 0.155259841 | 0.028226689 | 0.854000922 |
| cg17142470 | 0.032250475 | 9.6906534 | 1.21206544 | 77.47829467 |
| cg11795262 | 0.032276094 | 0.010838212 | 0.000172207 | 0.682124764 |
| cg26255848 | 0.032303125 | 0.096079783 | 0.011249481 | 0.820600032 |
| cg18762485 | 0.032358474 | 16815.86051 | 2.266848387 | 124742866.1 |
| cg07911673 | 0.032373828 | 0.161979163 | 0.030573553 | 0.858168157 |
| cg21517055 | 0.032381141 | 0.474873641 | 0.240060177 | 0.939368528 |
| cg22630748 | 0.032479228 | 0.427722193 | 0.196386066 | 0.931564433 |
| cg19290962 | 0.032488281 | 2.001089665 | 1.059576811 | 3.779206762 |
| cg21226234 | 0.03253532 | 0.055696192 | 0.003944263 | 0.786475443 |
| cg20227213 | 0.032573501 | 3.269948491 | 1.103297781 | 9.691457117 |
| cg09599653 | 0.032619878 | 9.333995894 | 1.202960968 | 72.42419471 |
| cg18888520 | 0.032797584 | 7299.832848 | 2.070077404 | 25741819.85 |
| cg04925864 | 0.032822312 | 0.051196517 | 0.003341075 | 0.784502971 |
| cg15350194 | 0.03288615 | 8.647144564 | 1.191768935 | 62.74128054 |
| cg02919422 | 0.032915125 | 50.62068919 | 1.375139211 | 1863.414376 |
| cg21119032 | 0.032930266 | 20389.9425 | 2.235984104 | 185935917.3 |
| cg07837085 | 0.032930444 | 0.53367292 | 0.29968602 | 0.950350589 |
| cg11719784 | 0.033007256 | 2.408981865 | 1.073520179 | 5.405761103 |
| cg19108718 | 0.033083561 | 0.506319063 | 0.270757427 | 0.946821647 |
| cg23492043 | 0.033138667 | 0.102979773 | 0.012719945 | 0.833716927 |
| cg22973042 | 0.033140932 | 0.142353835 | 0.023684275 | 0.855614741 |
| cg25692323 | 0.033244339 | 0.390309629 | 0.164164995 | 0.927978623 |
| cg16588061 | 0.033288931 | 39968410.53 | 4.001065619 | 3.99E+14 |
| cg25027167 | 0.033301624 | 0.120300023 | 0.017113068 | 0.84567509 |
| cg18182399 | 0.033303393 | 3.155271038 | 1.095201085 | 9.090326386 |
| cg06825166 | 0.033413553 | 2.043861493 | 1.057768716 | 3.949227974 |
| cg19771482 | 0.033539858 | 48.47351467 | 1.353047649 | 1736.584537 |
| cg13424229 | 0.03356555 | 1.815974946 | 1.047494866 | 3.148239777 |
| cg00782174 | 0.033585842 | 0.000329676 | 2.03E-07 | 0.536496385 |
| cg01120308 | 0.03359114 | 0.461553121 | 0.226210816 | 0.941737832 |
| cg15727249 | 0.0336903 | 0.25209343 | 0.070676926 | 0.899177435 |
| cg01939681 | 0.033748012 | 0.003513973 | 1.91E-05 | 0.64782088 |
| cg14182690 | 0.033753024 | 5.269338205 | 1.136136297 | 24.43890331 |
| cg03799530 | 0.033774922 | 0.003303806 | 1.69E-05 | 0.645273282 |
| cg11630392 | 0.033824579 | 0.142248189 | 0.023486844 | 0.861526889 |
| cg10617171 | 0.03388705 | 3.42E-06 | 3.05E-11 | 0.383747178 |
| cg23366752 | 0.033887064 | 0.426325299 | 0.193936951 | 0.937177054 |
| cg01043330 | 0.033977582 | 31.27456268 | 1.29744853 | 753.8628687 |
| cg01585703 | 0.034085183 | 31.89428962 | 1.296875516 | 784.3819226 |
| cg06090421 | 0.034131118 | 6.04E-05 | 7.55E-09 | 0.483329022 |
| cg25890048 | 0.034233297 | 0.52518087 | 0.289336452 | 0.953267187 |
| cg08108311 | 0.03423332 | 0.002362871 | 8.75E-06 | 0.637979115 |
| cg22082462 | 0.034272978 | 3.434061696 | 1.095744314 | 10.76234627 |
| cg16295988 | 0.034303384 | 42.46080079 | 1.319461454 | 1366.405663 |
| cg26964415 | 0.034308989 | 47086.87334 | 2.215369173 | 1000814522 |
| cg03041841 | 0.034322418 | 0.011167515 | 0.000173814 | 0.717512269 |
| cg23233234 | 0.034390289 | 13.21079259 | 1.208916032 | 144.364899 |
| cg24088438 | 0.034635354 | 0.255509689 | 0.072049128 | 0.906120624 |
| cg01861509 | 0.034665504 | 0.508483504 | 0.271474744 | 0.952410787 |
| cg08826839 | 0.03473137 | 0.493191523 | 0.255893581 | 0.950543102 |
| cg12099357 | 0.034744507 | 5.15E-07 | 7.48E-13 | 0.354150949 |
| cg21226224 | 0.034747119 | 80.06753444 | 1.369090893 | 4682.530652 |
| cg21461100 | 0.034939512 | 0.312534544 | 0.106048127 | 0.921070874 |
| cg27315279 | 0.035167246 | 4567992010 | 4.695228196 | 4.44E+18 |
| cg04533291 | 0.035181603 | 0.557328318 | 0.323487113 | 0.960207815 |
| cg03391568 | 0.035208986 | 0.368672707 | 0.145653895 | 0.933168073 |
| cg06479512 | 0.035215271 | 0.258884594 | 0.07359981 | 0.910616922 |
| cg21281001 | 0.035252515 | 1280.577265 | 1.639514268 | 1000221.935 |
| cg09325711 | 0.035333655 | 4957.347262 | 1.793950315 | 13698981.34 |
| cg06426831 | 0.035342539 | 0.051746207 | 0.003281233 | 0.816056078 |
| cg00626466 | 0.035365908 | 0.475510635 | 0.237926075 | 0.950338728 |
| cg21513553 | 0.03546329 | 0.114102744 | 0.015091147 | 0.862720133 |
| cg25750404 | 0.035463374 | 16334548136 | 4.95188784 | 5.39E+19 |
| cg12515371 | 0.03548672 | 0.426098888 | 0.192389067 | 0.943714034 |
| cg08784110 | 0.035521948 | 2.692065417 | 1.069374742 | 6.777059459 |
| cg14870461 | 0.035632136 | 0.228738128 | 0.057771293 | 0.905659687 |
| cg17982102 | 0.035634021 | 0.500527892 | 0.262448392 | 0.954580701 |
| cg02218324 | 0.036137121 | 0.563861397 | 0.329932874 | 0.963649578 |
| cg16334519 | 0.036264628 | 0.39959492 | 0.169328772 | 0.942994501 |
| cg02813863 | 0.036274407 | 0.029185055 | 0.001067713 | 0.797749792 |
| cg23173910 | 0.03638919 | 0.12756431 | 0.018540478 | 0.877682499 |
| cg04127255 | 0.036439537 | 0.291070398 | 0.091584706 | 0.92506686 |
| cg01888566 | 0.036456699 | 3.057061069 | 1.072965269 | 8.710088434 |
| cg12477119 | 0.036485508 | 0.074339569 | 0.006507546 | 0.849225124 |
| cg06134936 | 0.036625891 | 5.001068239 | 1.105264366 | 22.62868893 |
| cg14652095 | 0.03678508 | 0.469932742 | 0.23131458 | 0.954703255 |
| cg06276653 | 0.036795467 | 0.577689047 | 0.345146901 | 0.966906076 |
| cg03301801 | 0.03681181 | 0.493188132 | 0.253996833 | 0.957628217 |
| cg04711050 | 0.036813885 | 20845.07815 | 1.83863511 | 236326001 |
| cg13406768 | 0.036904158 | 0.203414509 | 0.045583492 | 0.907729106 |
| cg18419020 | 0.036928174 | 8797.75059 | 1.73503615 | 44610260.97 |
| cg10057218 | 0.036947406 | 0.296609508 | 0.094698399 | 0.929025214 |
| cg00145118 | 0.036966764 | 0.414125115 | 0.180891687 | 0.948079008 |
| cg15819853 | 0.037008216 | 4.44E-05 | 3.61E-09 | 0.5465779 |
| cg15534084 | 0.037090648 | 0.193627313 | 0.041363546 | 0.906390766 |
| cg11300809 | 0.037127362 | 5.388648952 | 1.105746249 | 26.26057971 |
| cg26381783 | 0.037131001 | 5547.460513 | 1.672567969 | 18399442.48 |
| cg25322008 | 0.037208227 | 0.462419195 | 0.223835177 | 0.955307898 |
| cg26044825 | 0.037232954 | 92.49142844 | 1.30710124 | 6544.760321 |
| cg06851207 | 0.037265712 | 0.38828887 | 0.159421785 | 0.945719226 |
| cg25974870 | 0.037295776 | 6013.583097 | 1.668733253 | 21671037.96 |
| cg05949660 | 0.037447247 | 0.497435098 | 0.257686338 | 0.960243678 |
| cg03521347 | 0.037521711 | 109.4087059 | 1.311328201 | 9128.35164 |
| cg09276883 | 0.037707827 | 0.021987696 | 0.000600538 | 0.805042505 |
| cg10031456 | 0.037805694 | 3.55177186 | 1.074003257 | 11.74585204 |
| cg25112191 | 0.037811725 | 3.818279394 | 1.078346436 | 13.52001272 |
| cg09190408 | 0.03799465 | 0.053633336 | 0.003382656 | 0.850377425 |
| cg04637372 | 0.03811093 | 1.698423474 | 1.02946854 | 2.802069404 |
| cg00864867 | 0.038128347 | 18290.12404 | 1.711303191 | 195481805.3 |
| cg09607282 | 0.038143354 | 0.027429906 | 0.000915862 | 0.821520615 |
| cg04784315 | 0.038184282 | 0.059312999 | 0.004103222 | 0.857382846 |
| cg15503752 | 0.03821395 | 3.376397579 | 1.068335082 | 10.6708661 |
| cg10848367 | 0.038245276 | 7.919315088 | 1.118618653 | 56.06517583 |
| cg05146762 | 0.038254125 | 289221.8787 | 1.975145826 | 42350946434 |
| cg07845392 | 0.038290742 | 0.457050906 | 0.217908034 | 0.958640793 |
| cg09182986 | 0.038334726 | 1.35E-05 | 3.33E-10 | 0.54747003 |
| cg24775607 | 0.038639953 | 0.448137694 | 0.20942762 | 0.958934608 |
| cg03173722 | 0.038651452 | 0.395733243 | 0.164367161 | 0.952774254 |
| cg27337148 | 0.038685948 | 41.5696815 | 1.213963309 | 1423.468408 |
| cg26394380 | 0.038700964 | 0.181861329 | 0.036135446 | 0.915265941 |
| cg10107186 | 0.038734025 | 0.071909508 | 0.005926151 | 0.872569327 |
| cg25658178 | 0.038841295 | 8173.303879 | 1.586928236 | 42095726.06 |
| cg05449607 | 0.038903217 | 0.000229867 | 8.10E-08 | 0.652495033 |
| cg23239039 | 0.039017351 | 65.07448707 | 1.234264593 | 3430.940895 |
| cg22449114 | 0.039314565 | 0.55323315 | 0.315068818 | 0.971428782 |
| cg22809047 | 0.039346815 | 0.508326053 | 0.267071943 | 0.967512249 |
| cg16173067 | 0.03940203 | 0.437268544 | 0.199038049 | 0.960639341 |
| cg07504780 | 0.03944064 | 5851903024 | 2.967053047 | 1.15E+19 |
| cg01980637 | 0.039466608 | 2.042533733 | 1.03504736 | 4.030679381 |
| cg03365354 | 0.03949462 | 6.03E-18 | 2.45E-34 | 0.148524449 |
| cg08084415 | 0.039567399 | 1459876.552 | 1.969347755 | 1.08E+12 |
| cg09261262 | 0.039697151 | 0.359482626 | 0.135610305 | 0.952934649 |
| cg10114327 | 0.039698888 | 1.807268457 | 1.028274352 | 3.176408387 |
| cg04368716 | 0.039909983 | 410417239.1 | 2.494866818 | 6.75E+16 |
| cg06766367 | 0.039917647 | 0.058310046 | 0.003875586 | 0.877302579 |
| cg18152712 | 0.039952197 | 25.53178974 | 1.160319386 | 561.8041851 |
| cg14817848 | 0.040051581 | 430.6171483 | 1.317148071 | 140782.2951 |
| cg13126790 | 0.040082333 | 2.426768362 | 1.040951177 | 5.657522482 |
| cg01182697 | 0.040084759 | 2.820742467 | 1.048052083 | 7.591786894 |
| cg13707560 | 0.040122179 | 4.395069031 | 1.069016654 | 18.06953307 |
| cg16379513 | 0.040123556 | 0.528182463 | 0.287119601 | 0.971639389 |
| cg12727374 | 0.040204342 | 7.14E-15 | 2.18E-28 | 0.233268355 |
| cg15076659 | 0.04025539 | 0.174847084 | 0.033034988 | 0.925428002 |
| cg04275985 | 0.040275847 | 1.80E+14 | 4.287224709 | 7.59E+27 |
| cg23246978 | 0.040335159 | 0.043013295 | 0.002125244 | 0.870555841 |
| cg08474603 | 0.040560931 | 0.560644524 | 0.322238222 | 0.975434512 |
| cg19007602 | 0.040574528 | 8.54E-11 | 1.97E-20 | 0.36973448 |
| cg06264060 | 0.040583507 | 3.891724448 | 1.059990307 | 14.28835629 |
| cg13878456 | 0.040635975 | 0.237812212 | 0.060125098 | 0.940616314 |
| cg05418129 | 0.040673616 | 0.211324989 | 0.047703919 | 0.936154769 |
| cg19616230 | 0.040715829 | 26.11099278 | 1.147762472 | 594.0113578 |
| cg19469297 | 0.040716191 | 8625.115878 | 1.466408367 | 50731178.01 |
| cg14588638 | 0.040779857 | 108.4587673 | 1.217186522 | 9664.339846 |
| cg07849904 | 0.040796063 | 9.85E-07 | 1.73E-12 | 0.560479892 |
| cg19328294 | 0.040887386 | 3525.439541 | 1.402662688 | 8860807.425 |
| cg14824983 | 0.040891689 | 0.411694977 | 0.175837112 | 0.963919117 |
| cg21057494 | 0.040925123 | 2.349308548 | 1.035859192 | 5.328186202 |
| cg01914242 | 0.040964489 | 4.52E-05 | 3.08E-09 | 0.663116226 |
| cg13359415 | 0.041020358 | 8967248.732 | 1.921512454 | 4.18E+13 |
| cg17054360 | 0.041092862 | 1405.309258 | 1.340710681 | 1473020.346 |
| cg08278554 | 0.04121827 | 0.202466337 | 0.043687097 | 0.938323223 |
| cg22130834 | 0.041269659 | 9174554884 | 2.481231107 | 3.39E+19 |
| cg05950276 | 0.041290426 | 31.79671493 | 1.146487023 | 881.8513079 |
| cg26025891 | 0.041413227 | 0.349172308 | 0.127019908 | 0.959859779 |
| cg13908523 | 0.041414974 | 1.63E+11 | 2.731842835 | 9.71E+21 |
| cg10269439 | 0.041461031 | 0.438609284 | 0.198614463 | 0.96860068 |
| cg19685976 | 0.041463435 | 4.94E-05 | 3.58E-09 | 0.681323841 |
| cg24560809 | 0.041552774 | 0.017673714 | 0.000364538 | 0.856865825 |
| cg15558658 | 0.041589418 | 3298093.212 | 1.771631126 | 6.14E+12 |
| cg13002506 | 0.041645199 | 0.545075722 | 0.304009018 | 0.977298453 |
| cg27248887 | 0.041689204 | 3.948110581 | 1.053038872 | 14.8024708 |
| cg03349935 | 0.041745239 | 0.158064779 | 0.026767613 | 0.933384468 |
| cg05373457 | 0.041785695 | 1.791529531 | 1.02191487 | 3.140748952 |
| cg22708853 | 0.041923761 | 0.006338633 | 4.83E-05 | 0.831202505 |
| cg00336605 | 0.041926893 | 0.006254761 | 4.71E-05 | 0.830860209 |
| cg22762180 | 0.04192979 | 273116091.1 | 2.032080623 | 3.67E+16 |
| cg07965839 | 0.041953728 | 16640057.76 | 1.83135301 | 1.51E+14 |
| cg19525717 | 0.042122372 | 0.031076325 | 0.001092761 | 0.883759826 |
| cg10334928 | 0.042158067 | 0.346362988 | 0.124559658 | 0.963131414 |
| cg03382304 | 0.042175811 | 0.011313749 | 0.000149973 | 0.853494985 |
| cg03112782 | 0.042255116 | 5146782.321 | 1.716872536 | 1.54E+13 |
| cg13620808 | 0.042414726 | 1.37E-05 | 2.75E-10 | 0.681592171 |
| cg09441152 | 0.042537347 | 7.45E-11 | 1.22E-20 | 0.456189195 |
| cg04959788 | 0.042550797 | 331.7525645 | 1.215291213 | 90562.4618 |
| cg03165700 | 0.042640364 | 469282.8499 | 1.542220322 | 1.43E+11 |
| cg25752527 | 0.042774405 | 1.48E-14 | 6.18E-28 | 0.354691719 |
| cg06245154 | 0.0428188 | 0.001502659 | 2.79E-06 | 0.810386404 |
| cg18383069 | 0.042907688 | 63.55621595 | 1.141754786 | 3537.881019 |
| cg12259537 | 0.042923419 | 2.461429532 | 1.02910941 | 5.887260653 |
| cg26979012 | 0.04294031 | 5533.609149 | 1.315049384 | 23284928 |
| cg20683151 | 0.042942695 | 77.22907361 | 1.148067208 | 5195.105102 |
| cg16050784 | 0.043030438 | 5652385541 | 2.022141439 | 1.58E+19 |
| cg06584407 | 0.043085436 | 0.313616628 | 0.101967426 | 0.964576561 |
| cg20804555 | 0.043119157 | 0.001079576 | 1.44E-06 | 0.809451908 |
| cg05348272 | 0.043135369 | 91.2842831 | 1.149526179 | 7248.91742 |
| cg14607011 | 0.043159089 | 0.497542034 | 0.252921218 | 0.978755671 |
| cg19928046 | 0.043166731 | 204.9678023 | 1.177678986 | 35673.38848 |
| cg22185378 | 0.043177449 | 9364647.506 | 1.63626453 | 5.36E+13 |
| cg02608292 | 0.043252055 | 6907.422887 | 1.307507011 | 36491193.2 |
| cg22230395 | 0.043363788 | 0.000289577 | 1.07E-07 | 0.78436761 |
| cg09099177 | 0.043493795 | 0.020658771 | 0.000477996 | 0.892862384 |
| cg11237817 | 0.043516106 | 0.470912642 | 0.226673265 | 0.978318801 |
| cg13460409 | 0.043546982 | 0.500247085 | 0.255318278 | 0.980138002 |
| cg05245861 | 0.043548573 | 11.3381376 | 1.072842145 | 119.8250506 |
| cg00480356 | 0.043573459 | 0.193691046 | 0.039335066 | 0.953760223 |
| cg27215108 | 0.043594996 | 20.55948098 | 1.090783309 | 387.5125836 |
| cg00821764 | 0.043616881 | 35.53232366 | 1.107671786 | 1139.819611 |
| cg16131766 | 0.043785779 | 0.534335999 | 0.290544235 | 0.982690155 |
| cg18997129 | 0.043926454 | 4.592275887 | 1.042354814 | 20.23207217 |
| cg21440587 | 0.044077562 | 0.399144325 | 0.163243853 | 0.975939913 |
| cg18062196 | 0.044159488 | 0.555698075 | 0.313579626 | 0.984758975 |
| cg23743472 | 0.044210859 | 0.548829502 | 0.305931817 | 0.984578277 |
| cg20781967 | 0.044303443 | 6.137582193 | 1.047315983 | 35.96805147 |
| cg10507231 | 0.044306266 | 2.27E-05 | 6.78E-10 | 0.761639294 |
| cg03440846 | 0.044379231 | 0.492655713 | 0.24706656 | 0.982365447 |
| cg07412254 | 0.044423272 | 2.446421322 | 1.022553449 | 5.85297257 |
| cg26908611 | 0.044444179 | 2.53E-08 | 9.89E-16 | 0.647655136 |
| cg02945646 | 0.04463997 | 0.203166194 | 0.042881622 | 0.96256859 |
| cg04561804 | 0.044725496 | 0.036033157 | 0.001404071 | 0.924731159 |
| cg02433671 | 0.044737103 | 3.613234991 | 1.030639699 | 12.6673435 |
| cg03843852 | 0.044823667 | 103.3871439 | 1.113089256 | 9602.915011 |
| cg05276137 | 0.044829696 | 3.94E-12 | 2.84E-23 | 0.545620446 |
| cg05168404 | 0.044870256 | 2.615359797 | 1.022245713 | 6.691255131 |
| cg18043455 | 0.044940425 | 0.000821701 | 7.93E-07 | 0.85188391 |
| cg01777397 | 0.044978461 | 0.291766592 | 0.087508415 | 0.972794956 |
| cg04134859 | 0.045000683 | 4.14E-17 | 3.98E-33 | 0.431347926 |
| cg22572159 | 0.045011464 | 7.606300398 | 1.046160365 | 55.30299911 |
| cg05140736 | 0.045081267 | 0.433155546 | 0.191096889 | 0.981825127 |
| cg13263114 | 0.045111762 | 0.087615836 | 0.008094688 | 0.948342263 |
| cg03343942 | 0.045149233 | 0.47834757 | 0.23249253 | 0.984188173 |
| cg26805528 | 0.045164093 | 1.801500113 | 1.012763067 | 3.204503365 |
| cg10732834 | 0.045177569 | 0.073906195 | 0.005776536 | 0.945571062 |
| cg26806924 | 0.045179616 | 0.176610127 | 0.032374397 | 0.963450754 |
| cg11375102 | 0.045188133 | 0.213507871 | 0.047119725 | 0.967442211 |
| cg04968426 | 0.045226298 | 0.195050276 | 0.039390055 | 0.965843031 |
| cg17217654 | 0.04522652 | 2454.190364 | 1.180525622 | 5102007.302 |
| cg15164103 | 0.045227969 | 9.347199146 | 1.048653473 | 83.31649505 |
| cg26656113 | 0.045269883 | 1.976980785 | 1.014460525 | 3.852740375 |
| cg04997967 | 0.045304047 | 2.572143993 | 1.019950143 | 6.486517762 |
| cg13277385 | 0.045312626 | 2.25E-13 | 9.31E-26 | 0.544559907 |
| cg27573888 | 0.045325676 | 2.69E-10 | 1.15E-19 | 0.632185695 |
| cg08136806 | 0.045367379 | 0.495154602 | 0.248757683 | 0.98561008 |
| cg04513422 | 0.045454131 | 0.392255491 | 0.156804709 | 0.981248401 |
| cg24844534 | 0.045464515 | 3.46E-08 | 1.69E-15 | 0.707013097 |
| cg18676053 | 0.045471922 | 0.512075053 | 0.265780544 | 0.986606679 |
| cg26490839 | 0.04553702 | 3142934.983 | 1.345799018 | 7.34E+12 |
| cg17067942 | 0.045687189 | 0.013494656 | 0.000197774 | 0.92077648 |
| cg26453670 | 0.045687553 | 0.347897985 | 0.123507599 | 0.979964058 |
| cg05480532 | 0.045838421 | 0.142719573 | 0.021115299 | 0.964650147 |
| cg00328227 | 0.045889738 | 0.492044535 | 0.245262376 | 0.987138051 |
| cg21666217 | 0.045940757 | 285.3717622 | 1.107272283 | 73547.44082 |
| cg04254916 | 0.046023928 | 2.187828793 | 1.013912279 | 4.720916127 |
| cg26780404 | 0.046160387 | 16607.48977 | 1.179990896 | 233738003.9 |
| cg07967308 | 0.046326815 | 0.026314772 | 0.000734718 | 0.942493593 |
| cg04711324 | 0.046339293 | 0.620406111 | 0.387896658 | 0.992284244 |
| cg05208878 | 0.046345798 | 4.234176233 | 1.023649292 | 17.51405341 |
| cg08241785 | 0.04639847 | 0.11055802 | 0.012660294 | 0.965465377 |
| cg00474209 | 0.046420905 | 0.001408834 | 2.20E-06 | 0.901128353 |
| cg16725865 | 0.046432747 | 770.9638124 | 1.110781689 | 535105.328 |
| cg20225681 | 0.046471765 | 0.108815724 | 0.012258548 | 0.965926941 |
| cg24272907 | 0.046498197 | 0.49911154 | 0.251811913 | 0.989279365 |
| cg12354377 | 0.046525541 | 0.195579393 | 0.039223908 | 0.975203668 |
| cg03495868 | 0.04653251 | 0.100528625 | 0.010468884 | 0.965337364 |
| cg01738359 | 0.046609258 | 7.94E-12 | 9.26E-23 | 0.681357526 |
| cg03266453 | 0.046669968 | 0.348835108 | 0.123589471 | 0.984597889 |
| cg07000831 | 0.04669662 | 4.19E-09 | 2.33E-17 | 0.754263331 |
| cg15679095 | 0.046720053 | 0.312759599 | 0.099482721 | 0.983271928 |
| cg10911877 | 0.046763687 | 29.9565058 | 1.049881497 | 854.7557434 |
| cg05253159 | 0.046772333 | 3.12184573 | 1.01638856 | 9.588774556 |
| cg13823701 | 0.046780059 | 0.567447037 | 0.324605498 | 0.991961451 |
| cg10222534 | 0.046814712 | 0.114664811 | 0.013555392 | 0.969947532 |
| cg16175792 | 0.046892123 | 1.772060669 | 1.007893429 | 3.115606197 |
| cg17240454 | 0.04698149 | 2.5248648 | 1.012433671 | 6.296651763 |
| cg07236190 | 0.0469927 | 0.544095924 | 0.298444957 | 0.991942963 |
| cg00692549 | 0.047016658 | 0.010412169 | 0.000115138 | 0.941591525 |
| cg08390209 | 0.047061473 | 0.277802059 | 0.07846814 | 0.983507245 |
| cg09273772 | 0.04715424 | 0.171807149 | 0.030178467 | 0.978104564 |
| cg06595693 | 0.047207574 | 0.446804311 | 0.201627124 | 0.990115258 |
| cg18113787 | 0.047226841 | 404559.9213 | 1.17126326 | 1.40E+11 |
| cg15757271 | 0.047316131 | 2.74E-08 | 9.26E-16 | 0.813633411 |
| cg27040030 | 0.047334293 | 4.162609566 | 1.016919582 | 17.03902521 |
| cg19724470 | 0.047342256 | 0.464210366 | 0.217439681 | 0.991039279 |
| cg20697204 | 0.047365028 | 0.153166368 | 0.023977357 | 0.978420435 |
| cg21008828 | 0.047393076 | 12.05163192 | 1.029045406 | 141.1422967 |
| cg18242139 | 0.047498472 | 0.535451543 | 0.288690971 | 0.993132393 |
| cg00576250 | 0.047518035 | 5.048507146 | 1.017879178 | 25.03973454 |
| cg17592292 | 0.047634795 | 846.4775636 | 1.072804747 | 667898.1124 |
| cg13992856 | 0.047636345 | 18.18596199 | 1.0306808 | 320.8842287 |
| cg15284635 | 0.047665768 | 0.542942259 | 0.296644255 | 0.993736745 |
| cg13637733 | 0.047678984 | 6390.290915 | 1.093766055 | 37335056.97 |
| cg14662756 | 0.047715579 | 0.079812465 | 0.006534205 | 0.974874467 |
| cg18895972 | 0.047846148 | 3.904865059 | 1.013004586 | 15.0522232 |
| cg11593656 | 0.04787357 | 0.091325943 | 0.00852944 | 0.97784007 |
| cg09553448 | 0.047996527 | 0.342665998 | 0.118534065 | 0.990601198 |
| cg00611397 | 0.048242152 | 0.119616086 | 0.014544751 | 0.983723153 |
| cg10481740 | 0.048310395 | 566.8670392 | 1.048208673 | 306559.4173 |
| cg16376234 | 0.048316551 | 761.0248585 | 1.050314078 | 551414.9026 |
| cg19394196 | 0.048450495 | 0.003241064 | 1.09E-05 | 0.961737885 |
| cg06284244 | 0.048510524 | 150422.0188 | 1.081098484 | 20929438047 |
| cg18885346 | 0.048510681 | 0.54263906 | 0.295636809 | 0.996009767 |
| cg00720137 | 0.048519959 | 20.00784505 | 1.019663848 | 392.5939558 |
| cg00141162 | 0.048546428 | 0.482954149 | 0.23433074 | 0.9953654 |
| cg19370284 | 0.04864307 | 0.471428878 | 0.223242774 | 0.995531379 |
| cg23614979 | 0.048677922 | 5.63E-07 | 3.44E-13 | 0.919899505 |
| cg14992108 | 0.048707347 | 0.498422539 | 0.249408129 | 0.996058262 |
| cg15911500 | 0.048773892 | 5.06E-23 | 3.38E-45 | 0.758718509 |
| cg00288562 | 0.048804208 | 26614.57904 | 1.054896083 | 671474497.5 |
| cg10920427 | 0.048974776 | 0.003492091 | 1.25E-05 | 0.974898516 |
| cg09584711 | 0.048995208 | 0.509456994 | 0.260318411 | 0.99703447 |
| cg06270401 | 0.049040366 | 0.366807322 | 0.135116242 | 0.995791548 |
| cg12688215 | 0.049154111 | 24938319.23 | 1.065135132 | 5.84E+14 |
| cg02658214 | 0.049208734 | 0.358108926 | 0.128699121 | 0.996448163 |
| cg02657438 | 0.049262534 | 0.458532212 | 0.210781748 | 0.997485752 |
| cg01464985 | 0.049276213 | 0.340534451 | 0.116360204 | 0.99659255 |
| cg15240064 | 0.049369155 | 0.114478093 | 0.013183881 | 0.994034592 |
| cg14264994 | 0.049562335 | 0.490192087 | 0.240616359 | 0.998636512 |
| cg06218044 | 0.04958883 | 1.45E-10 | 2.18E-20 | 0.960089275 |
| cg21053748 | 0.049597385 | 0.001514035 | 2.32E-06 | 0.988635935 |
| cg04970994 | 0.049621822 | 0.005823967 | 3.42E-05 | 0.991528931 |
| cg22968727 | 0.04966464 | 4.27E-05 | 1.85E-09 | 0.985359028 |
| cg11203041 | 0.049670638 | 2.764709683 | 1.001464994 | 7.632438153 |
| cg08448751 | 0.049672081 | 0.061254618 | 0.003767177 | 0.996005401 |
| cg04237003 | 0.049693474 | 0.489110025 | 0.239457919 | 0.999042413 |
| cg20202438 | 0.049712783 | 0.443273632 | 0.196692258 | 0.998979392 |
| cg00766729 | 0.049718173 | 186582.0213 | 1.015059041 | 34296380062 |
| cg09238598 | 0.049748176 | 42.79582902 | 1.004141765 | 1823.928697 |
| cg07034561 | 0.049801538 | 7.24E-08 | 5.31E-15 | 0.985847139 |
| cg25927124 | 0.049818097 | 2.55E-10 | 6.62E-20 | 0.982601409 |
| cg07868688 | 0.049932896 | 32009022.45 | 1.00507601 | 1.02E+15 |
| cg09462576 | 0.049949668 | 0.237651811 | 0.056496219 | 0.999684296 |
| cg00446235 | 0.049977655 | 1403.346154 | 1.000707093 | 1967988.876 |
