# Supplementary material for: DNA methylation subtypes for ovarian cancer prognosis
Source: FEBS Open Bio. 2021 Feb 3;11(3):851–65. doi: 10.1002/2211-5463.13056 (PMC7931230; doi:10.1002/2211-5463.13056)
Supplement: Supplementary file 2 — Table S2. 250 significant MET loci [file FEB4-11-851-s002.docx]

Table S2: 250 significant MET loci

|  | **Pvalue** |
| --- | --- |
| cg00431050 | 4.48E-05 |
| cg17407908 | 0.00034 |
| cg01804429 | 0.000627 |
| cg23111544 | 0.000675 |
| cg01278291 | 0.000708 |
| cg19342782 | 0.000725 |
| cg23910243 | 0.001082 |
| cg13727946 | 0.001147 |
| cg25620220 | 0.001872 |
| cg08946332 | 0.00196 |
| cg06392589 | 0.002027 |
| cg03770548 | 0.002063 |
| cg08044694 | 0.002158 |
| cg14672994 | 0.002287 |
| cg13064571 | 0.002529 |
| cg05859264 | 0.002576 |
| cg22283058 | 0.002675 |
| cg22467071 | 0.002763 |
| cg17808849 | 0.002867 |
| cg14378057 | 0.003073 |
| cg13263114 | 0.003197 |
| cg22784047 | 0.003364 |
| cg17169998 | 0.003502 |
| cg22381955 | 0.003679 |
| cg07000831 | 0.003794 |
| cg13273136 | 0.003805 |
| cg07026910 | 0.004411 |
| cg08013810 | 0.004521 |
| cg07236190 | 0.004571 |
| cg27299588 | 0.004651 |
| cg19108718 | 0.004678 |
| cg17976829 | 0.00471 |
| cg07260017 | 0.005473 |
| cg25634666 | 0.005486 |
| cg11599505 | 0.005553 |
| cg24983959 | 0.005657 |
| cg16949889 | 0.005713 |
| cg03259469 | 0.005772 |
| cg12920798 | 0.0058 |
| cg07603484 | 0.005953 |
| cg12312863 | 0.006084 |
| cg17543123 | 0.006133 |
| cg27305303 | 0.006284 |
| cg24735489 | 0.006308 |
| cg05955301 | 0.00646 |
| cg18382305 | 0.006564 |
| cg02357714 | 0.006624 |
| cg24237576 | 0.006778 |
| cg14226064 | 0.00686 |
| cg26776924 | 0.007056 |
| cg07559730 | 0.0072 |
| cg00579393 | 0.007343 |
| cg23146358 | 0.007638 |
| cg24088438 | 0.007816 |
| cg01044662 | 0.007853 |
| cg25169784 | 0.007878 |
| cg07892051 | 0.007911 |
| cg15020645 | 0.008127 |
| cg16725130 | 0.008186 |
| cg20576597 | 0.008332 |
| cg00294382 | 0.008338 |
| cg21461100 | 0.00851 |
| cg22991148 | 0.00894 |
| cg07967308 | 0.008977 |
| cg05252264 | 0.009406 |
| cg05443740 | 0.009469 |
| cg21581873 | 0.009655 |
| cg11456838 | 0.009893 |
| cg26806924 | 0.010027 |
| cg13098960 | 0.010087 |
| cg14799446 | 0.010261 |
| cg21023770 | 0.010477 |
| cg21207418 | 0.010481 |
| cg17055959 | 0.010491 |
| cg06607866 | 0.010556 |
| cg27637521 | 0.01064 |
| cg07753644 | 0.010955 |
| cg07519011 | 0.011048 |
| cg04706338 | 0.011115 |
| cg14947494 | 0.011273 |
| cg20202438 | 0.011307 |
| cg14163776 | 0.0116 |
| cg09234859 | 0.011679 |
| cg02657438 | 0.011853 |
| cg15942562 | 0.012054 |
| cg14056306 | 0.012472 |
| cg25123470 | 0.01254 |
| cg11203041 | 0.012895 |
| cg13878456 | 0.013014 |
| cg27488807 | 0.013179 |
| cg00769470 | 0.013197 |
| cg06840239 | 0.014019 |
| cg13620808 | 0.014062 |
| cg15534084 | 0.014375 |
| cg05836145 | 0.015191 |
| cg25322008 | 0.015342 |
| cg26656113 | 0.015687 |
| cg02539714 | 0.015791 |
| cg09099177 | 0.015999 |
| cg15643724 | 0.016102 |
| cg03568064 | 0.016231 |
| cg11473104 | 0.016252 |
| cg04457051 | 0.016488 |
| cg21291896 | 0.016928 |
| cg12970081 | 0.017031 |
| cg22968727 | 0.017479 |
| cg12100791 | 0.017559 |
| cg09273772 | 0.017979 |
| cg27210136 | 0.018397 |
| cg06529761 | 0.018471 |
| cg11177693 | 0.018629 |
| cg06095560 | 0.018768 |
| cg23898073 | 0.018812 |
| cg22708853 | 0.019025 |
| cg22215192 | 0.019083 |
| cg23486067 | 0.019117 |
| cg05316065 | 0.019312 |
| cg25927124 | 0.019321 |
| cg16114640 | 0.01935 |
| cg20227213 | 0.019361 |
| cg03257423 | 0.019412 |
| cg13060646 | 0.019512 |
| cg07837085 | 0.019637 |
| cg10334928 | 0.019746 |
| cg00520708 | 0.020127 |
| cg03173722 | 0.020395 |
| cg09599653 | 0.020683 |
| cg27022827 | 0.021474 |
| cg06584407 | 0.021576 |
| cg19464252 | 0.021613 |
| cg18641050 | 0.022003 |
| cg06178072 | 0.022052 |
| cg26908611 | 0.022127 |
| cg18910313 | 0.022575 |
| cg21742836 | 0.022593 |
| cg26025891 | 0.02263 |
| cg10484958 | 0.022665 |
| cg22197830 | 0.022714 |
| cg06391660 | 0.02286 |
| cg00554250 | 0.023008 |
| cg12188560 | 0.023137 |
| cg11300809 | 0.023249 |
| cg09156233 | 0.023435 |
| cg17285325 | 0.023508 |
| cg18419020 | 0.023971 |
| cg06851207 | 0.02399 |
| cg06291334 | 0.024395 |
| cg20543571 | 0.02447 |
| cg09607282 | 0.024647 |
| cg00141162 | 0.025456 |
| cg02144933 | 0.025755 |
| cg06479512 | 0.025995 |
| cg10940099 | 0.026057 |
| cg09182986 | 0.026192 |
| cg07576541 | 0.026393 |
| cg00782174 | 0.02663 |
| cg01275830 | 0.026722 |
| cg03693099 | 0.026766 |
| cg03156547 | 0.026854 |
| cg00655307 | 0.026897 |
| cg19394196 | 0.026993 |
| cg01777397 | 0.027169 |
| cg12966875 | 0.027261 |
| cg15271616 | 0.027276 |
| cg01990225 | 0.027396 |
| cg16547341 | 0.027397 |
| cg01414934 | 0.027661 |
| cg00145118 | 0.028369 |
| cg06245154 | 0.028655 |
| cg26626042 | 0.028764 |
| cg27281093 | 0.029398 |
| cg17384145 | 0.0294 |
| cg22862656 | 0.029405 |
| cg16022344 | 0.029529 |
| cg17274742 | 0.029832 |
| cg05656364 | 0.029901 |
| cg00336605 | 0.030056 |
| cg26608667 | 0.031203 |
| cg10107186 | 0.031358 |
| cg13823701 | 0.031406 |
| cg18414381 | 0.031683 |
| cg21281799 | 0.031719 |
| cg15339605 | 0.032174 |
| cg09459044 | 0.032392 |
| cg17718515 | 0.032808 |
| cg15911500 | 0.03296 |
| cg18043455 | 0.033063 |
| cg08936952 | 0.033289 |
| cg05869585 | 0.03339 |
| cg02537838 | 0.033412 |
| cg10073091 | 0.033781 |
| cg25752527 | 0.034325 |
| cg03375833 | 0.034563 |
| cg12477119 | 0.035142 |
| cg13084525 | 0.035239 |
| cg01110312 | 0.035543 |
| cg12087643 | 0.035621 |
| cg00672638 | 0.035956 |
| cg13033054 | 0.036082 |
| cg25044651 | 0.036141 |
| cg16723180 | 0.036251 |
| cg04254119 | 0.036298 |
| cg17950095 | 0.036631 |
| cg05959508 | 0.037017 |
| cg07906724 | 0.037021 |
| cg15819853 | 0.037128 |
| cg02992632 | 0.03732 |
| cg05322019 | 0.037468 |
| cg24888049 | 0.03757 |
| cg14377791 | 0.037641 |
| cg05146762 | 0.037682 |
| cg03752628 | 0.037896 |
| cg06276653 | 0.038048 |
| cg25263140 | 0.038656 |
| cg05130485 | 0.039491 |
| cg08338368 | 0.040334 |
| cg01656216 | 0.040414 |
| cg21440587 | 0.040416 |
| cg15652212 | 0.040433 |
| cg19616230 | 0.041271 |
| cg07952391 | 0.041286 |
| cg24332422 | 0.042223 |
| cg19298821 | 0.042565 |
| cg22809047 | 0.042605 |
| cg11237817 | 0.042665 |
| cg06797533 | 0.042694 |
| cg06059810 | 0.043064 |
| cg26428825 | 0.04311 |
| cg16173067 | 0.043667 |
| cg00910067 | 0.043925 |
| cg09503974 | 0.044214 |
| cg15425811 | 0.04446 |
| cg06638012 | 0.044802 |
| cg15448599 | 0.046369 |
| cg08784110 | 0.046607 |
| cg20807701 | 0.046858 |
| cg09276883 | 0.046975 |
| cg04663487 | 0.047065 |
| cg18997129 | 0.047083 |
| cg00328227 | 0.047349 |
| cg02100848 | 0.04753 |
| cg01464985 | 0.047611 |
| cg04637372 | 0.047641 |
| cg03152385 | 0.048273 |
| cg27625732 | 0.048453 |
| cg23696886 | 0.048488 |
| cg22916109 | 0.048635 |
| cg25829729 | 0.049236 |
| cg23173910 | 0.049556 |
| cg09988116 | 0.049692 |
