# Supplementary material for: DNA methylation subtypes for ovarian cancer prognosis
Source: FEBS Open Bio. 2021 Feb 3;11(3):851–65. doi: 10.1002/2211-5463.13056 (PMC7931230; doi:10.1002/2211-5463.13056)
Supplement: Supplementary file 3 — Table S3. Distribution of methylation levels in each subtype [file FEB4-11-851-s003.docx]

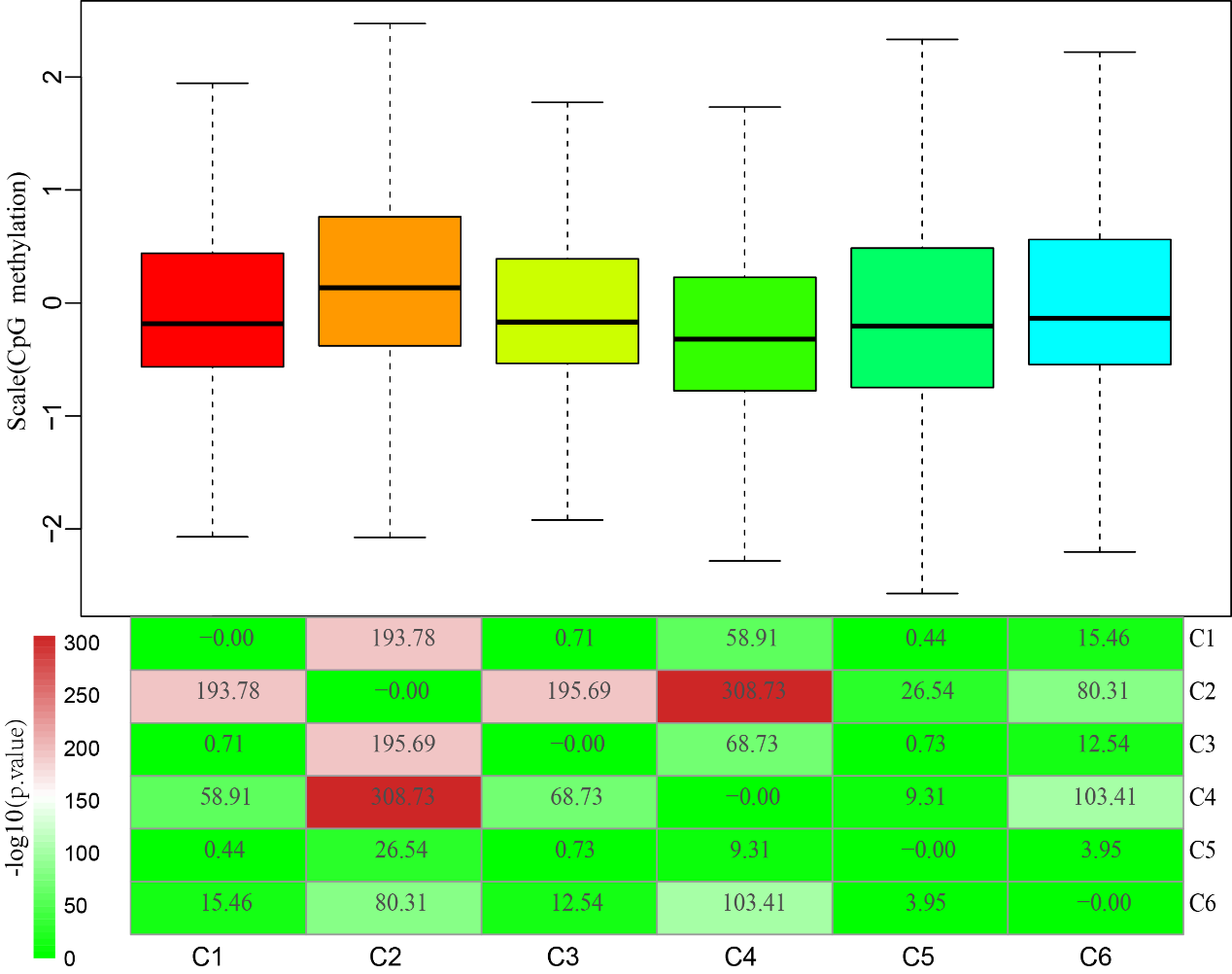


Table Supp:3 distribution of methylation levels in each subtype. T.test was used to examine the pantwise subtypes. Significant methylation differences were observed between most subtypes. C2 subtype was significantly different from the other five subtypes, p<0.01.
