# Supplementary material for: DNA methylation subtypes for ovarian cancer prognosis
Source: FEBS Open Bio. 2021 Feb 3;11(3):851–65. doi: 10.1002/2211-5463.13056 (PMC7931230; doi:10.1002/2211-5463.13056)
Supplement: Supplementary file 4 — Table S4. The distribution of grade and stage in each subtype [file FEB4-11-851-s004.docx]

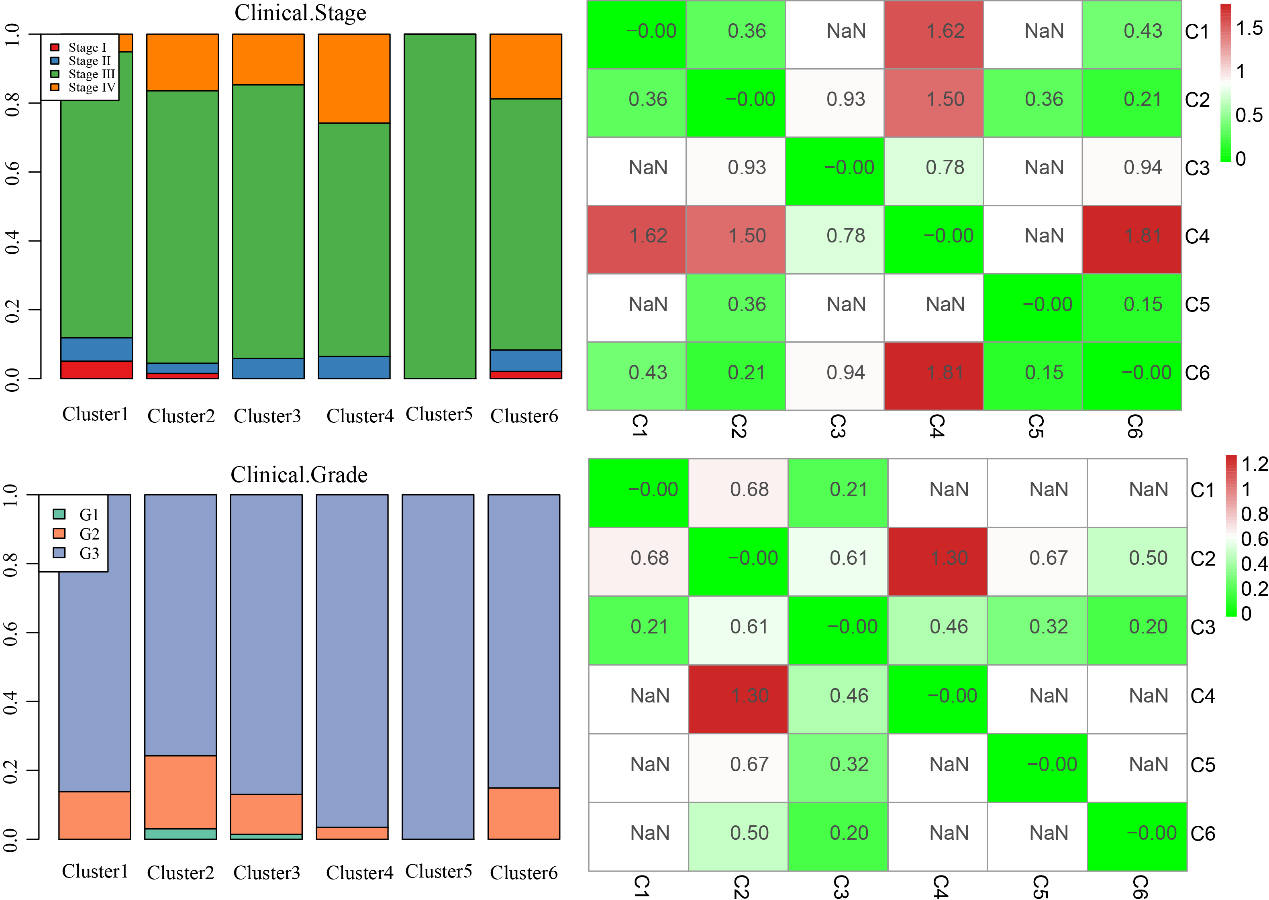


Table Supp4: The distribution of Grade and Stage in each subtype, and the chi-square test is used to examine the subtypes between the two.
