# Supplementary material for: DNA methylation subtypes for ovarian cancer prognosis
Source: FEBS Open Bio. 2021 Feb 3;11(3):851–65. doi: 10.1002/2211-5463.13056 (PMC7931230; doi:10.1002/2211-5463.13056)
Supplement: Supplementary file 5 — Table S5. Age distribution of samples in each subtype [file FEB4-11-851-s005.docx]

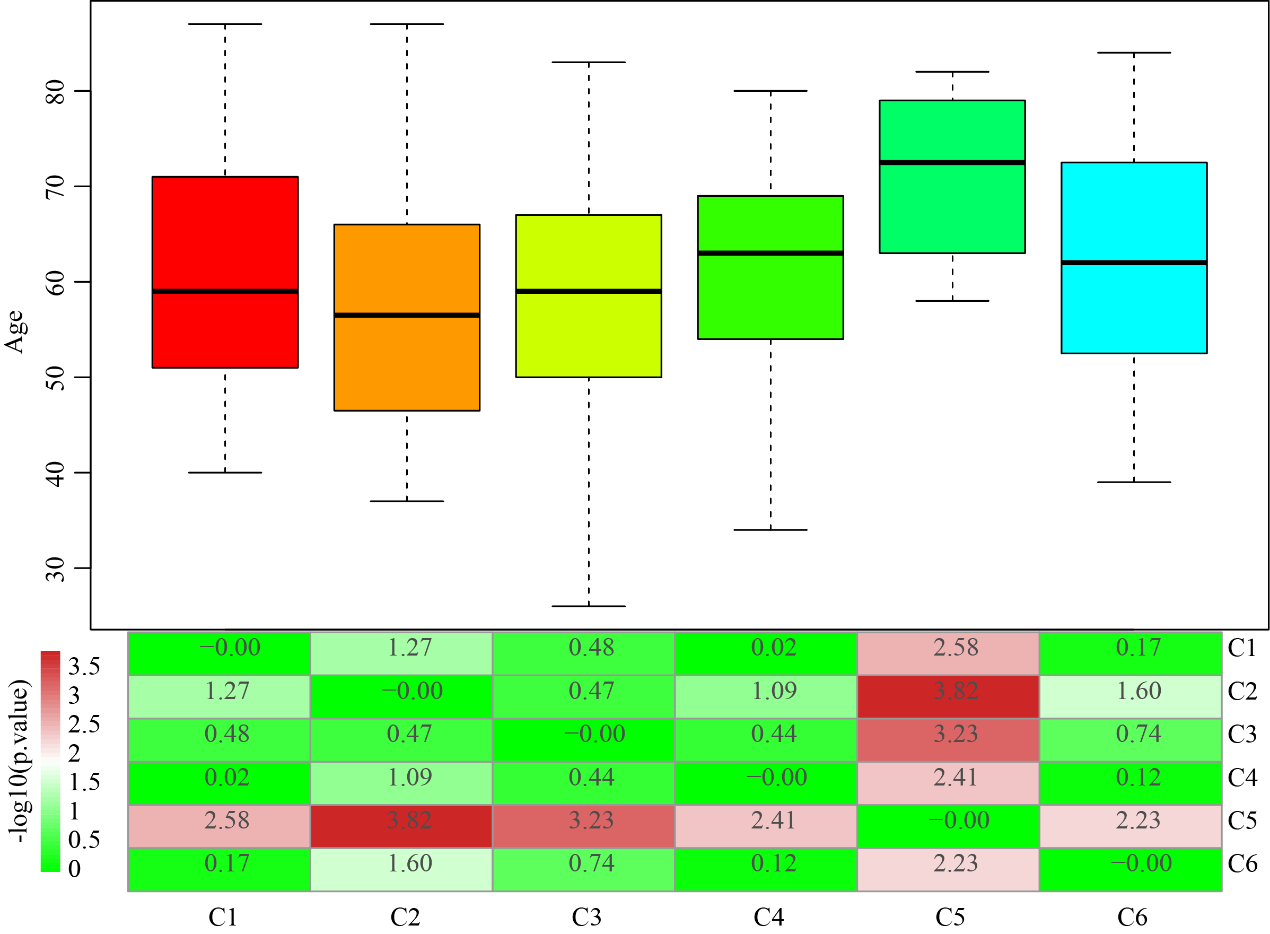


Table Supp5: Age distribution of samples in each subtype,

T.test was used to test the subtypes between the two. It can be seen that the age of C5 subtype is significantly higher than that of other groups, with an average p<0.001.
