# Supplementary material for: DNA methylation subtypes for ovarian cancer prognosis
Source: FEBS Open Bio. 2021 Feb 3;11(3):851–65. doi: 10.1002/2211-5463.13056 (PMC7931230; doi:10.1002/2211-5463.13056)
Supplement: Supplementary file 6 — Table S6. The R package covered in this article [file FEB4-11-851-s006.docx]

Table S6: The R package covered in this article

| **1. Impute** | Used for missing value completion |
| --- | --- |
|  |  |
| **2. Survival** | Survival analysis |
|  |  |
| **3. ConcensusClusterPlus** | Consistent clustering |
|  |  |
| **4. WGCNA** | Co-expression network |
|  |  |
| **5. Pheatmap** | Draw heatmap, cluster analysis |
